# Feasibility study on the use of “Qi-tonifying medicine compound” as an anti-fatigue functional food ingredient based on network pharmacology and molecular docking

**DOI:** 10.3389/fnut.2023.1131972

**Published:** 2023-05-05

**Authors:** Yi Wu, Yixuan Ma, Jinguo Cao, Rui Xie, Feng Chen, Wen Hu, Yushan Huang

**Affiliations:** ^1^Center for Evidence Based Medical and Clinical Research, First Affiliated Hospital of Gannan Medical University, Ganzhou, China; ^2^Key Laboratory of Prevention and Treatment of Cardiovascular and Cerebrovascular Diseases, Ministry of Education, Gannan Medical University, Ganzhou, China; ^3^Jiangxi Province Key Laboratory of Biomaterials and Biofabrication for Tissue Engineering, Gannan Medical University, Ganzhou, China; ^4^College of Pharmacy, Gannan Medical University, Ganzhou, China; ^5^School of Basic Medical Sciences, Gannan Medical University, Ganzhou, China; ^6^Department of Pediatric Surgery, The First Affiliated Hospital of GanNan Medical University, Ganzhou, China

**Keywords:** *Eucommia ulmoides* Oliver bark, *Eucommia ulmoides* Oliver male flower, *Panax notoginseng*, *Syzygium aromaticum*, anti-fatigue, herbal compound, functional food, network pharmacology

## Abstract

**Introduction:**

Fatigue has attracted broad attention in recent years due to its high morbidity rates. The use of functional foods to relieve fatigue-associated symptoms is becoming increasingly popular and has achieved relatively good results. In this study, network pharmacology and molecular docking strategies were used to establish the material basis and mechanisms of Chinese herbal compounds in fatigue treatment. According to traditional medicine theories and relevant guidance documents published by the Chinese Ministry of Health, four herbal medicines, including *Eucommia ulmoides* Oliver bark, *Eucommia ulmoides* Oliver male flower, *Panax notoginseng*, and *Syzygium aromaticum* (EEPS), were selected to constitute the anti-fatigue herbal compound that may be suitable as functional food ingredients.

**Methods:**

The major active ingredients in EEPS were identified via comprehensive literature search and Traditional Chinese Medicine Systems Pharmacology database search. Corresponding targets for these ingredients were predicted using SwissTargetPrediction. The network was constructed using Cytoscape 3.9.1 to obtain key ingredients. Prediction of absorption, distribution, metabolism, excretion and toxicity properties was performed using the ADMETIab 2.0 database. The anti-fatigue targets were retrieved from GeneCards v5.13, OMIM, TTD and DisGeNET 7.0 databases. Then, the potential targets of EEPS in fatigue treatment were screened through a Venn diagram. A protein–protein interaction (PPI) network of these overlapping targets was constructed, and the hub targets in the network selected through topological screening. Gene Ontology and KEGG pathway enrichment analyses were performed using the DAVID database and the bioinformatics online platform. Finally, AutoDock tools were used to verify the binding capacity between the key active ingredients and the core targets.

**Results and Discussion:**

This study identified the active ingredients and potential molecular mechanisms of EEPS in fatigue treatment, which will provide a foundation for future research on applications of herbal medicines in the functional food industry.

## 1. Introduction

Fatigue is a common and complex psychophysiology disease involving multiple factors (1–3). Given aging of the global population and the accelerating pace of life, fatigue incidences are increasing annually (4). Fatigue is an early signal of related diseases but can also be the sequelae of multiple complex diseases. The World Health Organization (WHO) has declared that fatigue is a major risk factor for human life and health (5). The COVID-19 pandemic aggravated the risk of fatigue (6, 7). A large study involving more than 40,000 patients confirmed that a majority of cases (> 80%) presented at least one symptom 4 weeks after being diagnosed (8). Among the symptoms, fatigue was the most common, occurring in up to 58% of patients.

Since fatigue is a chronic and complex disease, there is a need to exploit novel multitarget therapies for long-term use (9). Functional foods were first introduced in Japan in 1984 (10). This class of foods is capable of providing nutrition but can also play a role in diabetes prevention and treatment (11). With rapid advances of the food industry and scientific research, the scope of functional foods has been iteratively expanded to include foods with therapeutic, prophylactic, and nutritive properties (10). Compared with conventional drugs, functional foods have a higher safety profile and higher adherence. The functional foods market has rapidly increased, attracting attention from consumers and merchants (12). Thus, designing novel functional foods has become a topic of focus.

Traditional herbals have a storied history of clinical use, and they are known for their characteristics of “multicomponents, multitargets and multipathways” (13–18). Therefore, they have attracted increasing research attention. Due to their well-defined efficacy and excellent safety, some herbal medicines act as drugs and can be used as raw materials in functional foods. According to characteristics of TCM-defined syndromes, fatigue is primarily driven by ‘xuzheng’. Therefore, the use of tonifying medicines can exert the effects of reinforcing deficiency and ‘fu zheng’, leading to improved fitness and enhanced anti-fatigue abilities (19, 20). Due to the large number of ‘Qi-tonifying medicines’, it is necessary to develop a strategy for identifying the most potent anti-fatigue medicines with low toxicity that are suitable for long-term administration. By 2020, more than 200 varieties of TCM were approved as medicine and food resources by the Chinese Ministry of Health (21–28). Throughout the years of application, these TCMs were used as major constituents in pharmaceutical formulations, and are considered safe and effective food additives. Among these TCMs, several classical tonifying medicines, including *Eucommia ulmoides* Oliver bark (EUOB)*, Eucommia ulmoides* Oliver male flowe*r* (EUOF), *Panax notoginseng* (PN), and *Syzygium aromaticum* (SA), have attracted our research interests and attention (29–32). The traditional medicine theory postulates that the herbal combination has better therapeutic efficacies than single herbs by rational design (33–35). This theory is widely accepted and has become an important research tool for designing novel therapeutics for multiple diseases, such as digestive, neurological, and respiratory diseases (15, 33). Several types of functional foods that use these tonifying medicines or their combinations as the main raw materials have been developed, with satisfactory results (36–40). Among these products, EUOB has the highest frequency and is often used as the main component of related products. Traditionally, EUOB is thought to be a major medicinal component for prevention and treatment of chronic diseases such as osteoporosis, arthritis, and hypertension (41–43). However, slow growth of trunk bark has limited the supply of EUOB. In addition, peeling off too much bark is likely to lead to the death of EUO trees. To ensure optimal use of EUO resources, studies have aimed at establishing the significance of other medicinal parts of EUO (42, 44). Even though EUOF and EUOB have comparable compositions, there are some differences in contents of active ingredients between them. For instance, the amounts of flavone compounds in EUOF are much higher than those in EUOB. Flavonoids have antioxidant, anti-inflammatory, and other properties that are anti-fatigue. This shows that EUOF is a potential material basis for sustainable applications of EUOB (45). Some merchants have already used the EUOF in form of tea as novel functional foods. These products have received positive feedbacks from consumers. Therefore, utility of EUOF as the raw material in functional foods or as an anti-fatigue agent is a research direction that is worthy of further exploration. PN is widely distributed in Southwest China, Nepal, and Myanmar and has been commonly used as a traditional medicine for a long time (46). The *Panax notoginseng* saponins (PNS) are the main active ingredients in PN (47). Lin et al. reported that combining PNS with other types of herbs can improve their biological activities (48). Therefore, PN is a good candidate as an ideal ingredient in composite prescriptions of TCM. SA (cloves) are aromatic dried flower buds of the Myrtaceae family. They have been used in China for many years and are regarded as being among the most potent medicinal herbs. SA and its component (eugenol) have various biological functions, including antioxidant and anti-inflammatory activities (49). Supplementation of SA can improve the flavor quality of foods and exert antioxidant as well as antimicrobial effects (50). Therefore, SA is a potential pharmacodynamic constituent and natural preservative in functional food formulations. Based on the above findings, we propose that combination prescriptions composed of ‘EUOB, EUOF, PN and SA can exert anti-fatigue effects.

Due to complicated constituents of medicinal herbs, it is challenging to establish their mechanisms of action. To overcome this challenge, Li et al. proposed a network pharmacology strategy (51). Through comprehensive applications of pharmacology and systems biology, combining polypharmacology, bioinformatics, and computer simulations, network pharmacology can systematically explain the multicomponent and multitarget mechanisms of action of traditional Chinese medicines (52, 53). Molecular docking is also an efficient tool for analyzing the affinity, activity, and binding modes of ligands with target proteins (54, 55). In this study, we adopted network pharmacology and molecular docking to assess the anti-fatigue mechanisms of EEPS and to provide a theoretical basis for applications of EEPS as novel functional food additives.

## 2. Materials and methods

### 2.1. Collection of active compounds and potential targets of EEPS

Most of the active compounds of EUOB, PN and SA were obtained from the Traditional Chinese Medicine Systems Pharmacology Database and Analysis Platform v2.3 (TCMSP v2.3) database and screened by oral bioavailability (OB ≥ 30%) and drug likeness (DL ≥ 0.18) (56–58). To comprehensively establish the pharmacodynamic ingredients of EEPS, some active compounds were added according to the search results of the four databases, ZhiWang, WeiPu, and WanFang. The TCMSP v2.3 database does not provide any information on EUOF. The active compounds of EUOF were only obtained via an extensive literature search. All the active compounds were collated by TCMSP v2.3. Then, targets of active ingredients in EEPS were collected from TCMSP v2.3, and their official gene symbols converted using the UniProt database (59). The specific conversion process was performed as follows. Target protein names for each herb were collated from TCMSP v2.3 and imported into the UniProtKB module of the UniProt database, with the status set as “Reviewed” and popular organisms set as “Human.” Then, we converted the potential target protein names to their official gene symbol names. Targets of active ingredients were also searched in SwissTargetPrediction databases (60) with *Homo sapiens*. Then, all potential targets of EEPS, as predicted by the two databases, were merged and duplicates were removed to obtain the targets of EEPS. Each herbal medicine in EEPS has common and unique active compounds or targets. For the convenience of readers, active compounds and targets for each herbal medicine were separately imported into the EVenn online platform to plot the intersection of compounds and intersection of targets in the Venn diagram.

### 2.2. Identification of anti-fatigue targets

A comprehensive search was conducted in multiple databases [Online Mendelian Inheritance in Man (OMIM) (61), Therapeutic Target Database (TTD) (62) and DisGeNET 7.0 (63) databases] with the keywords “fatigue” and “anti fatigue.” All the targets from GeneCards v5.13 (64), OMIM, TTD and DisGeNET 7.0 databases were combined, and duplicates were removed to obtain fatigue-related targets. To clarify the anti-fatigue targets of EEPS, targets of EEPS and fatigue-related targets were entered into the EVenn online platform (65). The overlapping targets were considered to be the potential targets for EEPS against fatigue.

### 2.3. Construction of the H-A-T network

To understand the complex interactions between herbal medicines, active compounds, and corresponding targets, the herbal medicine-active compound-targetnetwork (H-A-T) was constructed using the Cytoscape 3.9.1 (66) software. Crucial nodes with high degrees were screened using the “Analyze Network” function, thereby obtaining the key anti-fatigue compounds.

### 2.4. ADMET (absorption, distribution, metabolism, excretion, and toxicity) analysis of key components

Structures (SMILES format) of active compounds were separately imported into the ADMETIab 2.0 database (67). Octanol/water partition coefficient (logP), number of hydrogen bond donors (HBD), number of hydrogen bond acceptors (HBA) and other physicochemical properties were obtained using the ADMET Screening function of this database.

### 2.5. Construction of the PPI network

The STRING 11.5 database (68) was used to construct a protein–protein interaction (PPI) network for the activities of EEPS against fatigue. The biological species was set as “*Homo sapiens*,” the minimum interaction threshold was set to “0.4,” while the rest of the parameters were kept at default to obtain the PPI network. Then, visualization and analysis of the PPI network were performed using Cytoscape 3.9.1. Ultimately, the top ranked targets by network degree were selected as the key anti-fatigue targets.

### 2.6. Go and KEGG pathway enrichment analyses

The DAVID v2022 q2 database was used for Gene Ontology (GO) and Kyoto Encyclopedia of Genes and Genomes (KEGG) pathway enrichment analyses (69). The organism was set to *Homo sapiens*, and “Gene list” was selected as the list type. Terms enriched with *p* < 0.01 and enrichment factor > 1.5 were considered significant. The top 10 most significantly enriched biological process (BP), cellular component (CC), and molecular function (MF) items and top 20 KEGG terms were selected. The bioinformatics online platform was used for data visualization (70).

### 2.7. Molecular docking

Crystal structures of key targets were obtained from the UniProt and RCSB PDB protein databases and saved in the PDB format (71). Small molecule ligands and water molecules were removed using AutoDock 4.0. The 3D structures of key components were obtained from the PubChem database (72). Then, Autodock Tools was used to prepare the receptor file, including adding Gasteiger charges, merging nonpolar hydrogen atoms, and preparing a pdbqt file. Finally, analysis of interactions between targets and key components was performed although flexible docking using the AUTOGRID program (73).

**Figure 1:**
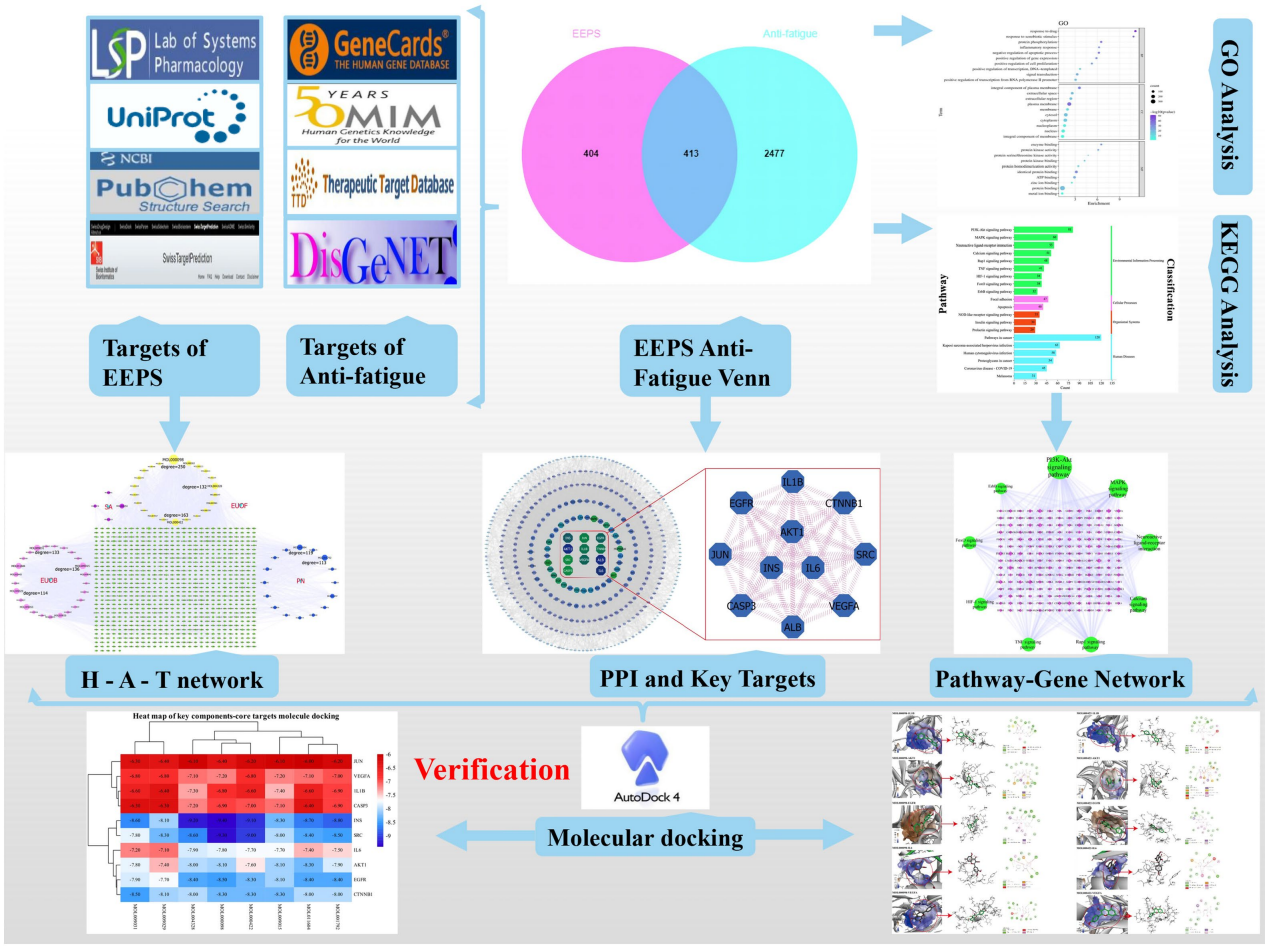
Flow diagram of this study.

Enzyme structure inputs were processed, and flexible ligand docking performed using the AUTOGRID program, with other values set as default parameters. The result of docking is the binding energy; the smaller the binding energy, the more stable the ligand and receptor are bound and the more likely the interactions are to occur (Figure 1).

## 3. Results

### 3.1. Active compounds of EEPS and their potential targets

**Figure 2:**
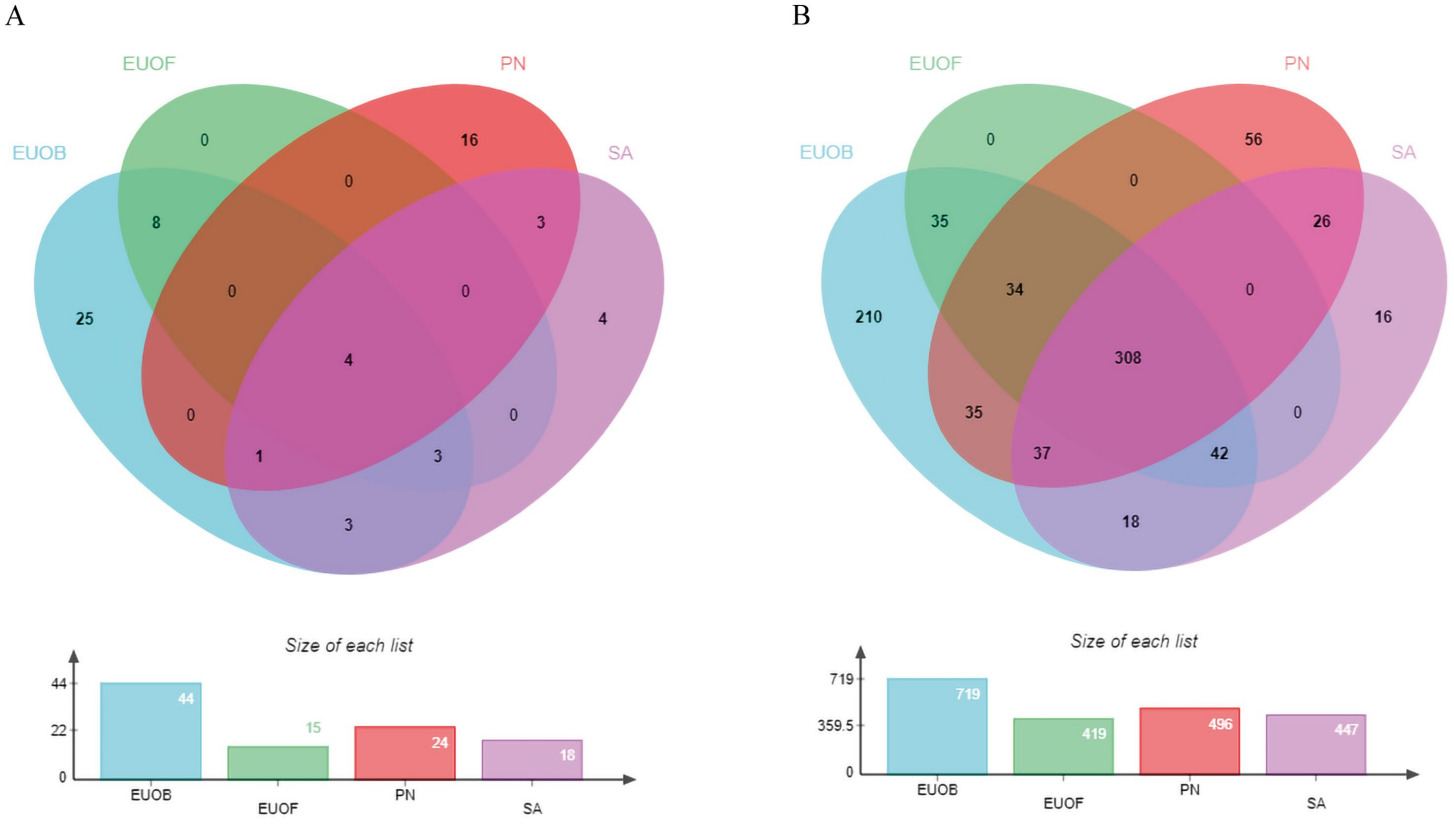
Venn diagrams of active components and predicted targets by TCMSP v2.3, UniProt and SwissTargetPrediction (blue for EUOB, green for EUOF, red for PN, and pink for SA). **(A)** Venn diagram of active components; **(B)** Venn diagram of potential predicted targets for each herb of EEPS.

Sixty seven active compounds of EEPS were collected from the TCMSP v2.3 database and from other search methods (Supplementary Table S1). In Supplementary Table S1, even though the OB and DL values for chlorogenic acid, geniposide, geniposidic acid, rutin, caffeic acid, and ginsenoside rb1 are not yet eligible for screening, there is a large body of literature that addresses their relations to fatigue. Thus, these compounds were also included in candidate compounds for further analyses so that more accurate results could be obtained. After prediction and screening by multiple online databases (TCMSP v2.3, UniProt and SwissTargetPrediction databases), 817 potential targets of the 126 components were identified. All active compounds of EUOB, EUOF, PN and SA and their corresponding targets were imported into the EVenn online platform to analyze intersections of the datasets (Figure 2). In Figure 2A, there were 4 intersections between components of EUOB, EUOF, PN and SA; 8 intersections between the components of EUOB and EUOF; 3 intersections between the components of EUOB and SA; 3 intersections between the components of PN and SA; 3 intersections between the components of EUOB, EUOF and SA, and 1 intersection between the components of EUOB, PN and SA. In Figure 2B, there were 308 intersections between targets of EUOB, EUOF, PN and SA; 35 intersections between targets of EUOB and EUOF; 35 intersections between the targets of EUOB and PN; 18 intersections between the targets of EUOB and SA; 26 intersections between the targets of PN and SA; 34 intersections between the targets of EUOB, EUOF and PN; 42 intersections between the targets of EUOB, EUOF and SA, and 37 intersections between the targets of EUOB, PN and SA. These findings indicate that the four herbs have some shared active components and bioactive targets. However, these herbs also have their own characteristics.

### 3.2. Fatigue targets of the main compounds from EEPS

**Figure 3:**
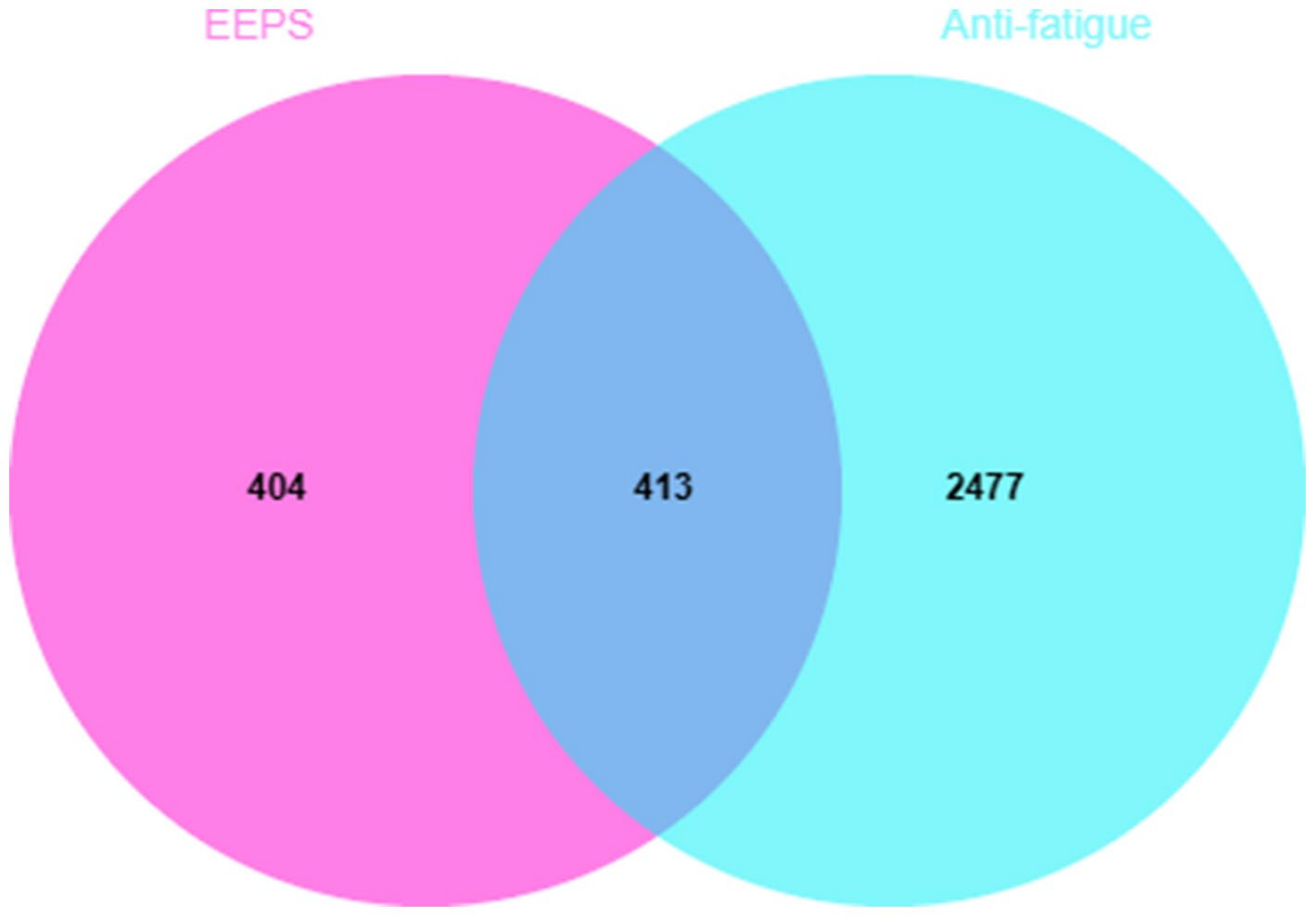
Venn diagram of EEPS for fatigue: the pink part indicates the potential targets of EEPS, the light blue part indicates the disease targets of fatigue, while the dark blue part indicates the overlapping targets.

After searching the GeneCards v5.13, OMIM, TTD and DisGeNET 7.0 databases, 2,890 potential fatigue targets were obtained after removing duplicate targets. Then, the 817 predicted targets of the active compounds of EEPS and the 2,890 fatigue-related disease targets were imported into the EVenn online platform to construct the Venn diagram. Visual results are shown in Figure 3. A total of 413 overlapping targets of EEPS with potential anti-fatigue effects were obtained for subsequent analyses.

### 3.3. Construction of H-A-T network and key component selection

**Figure 4:**
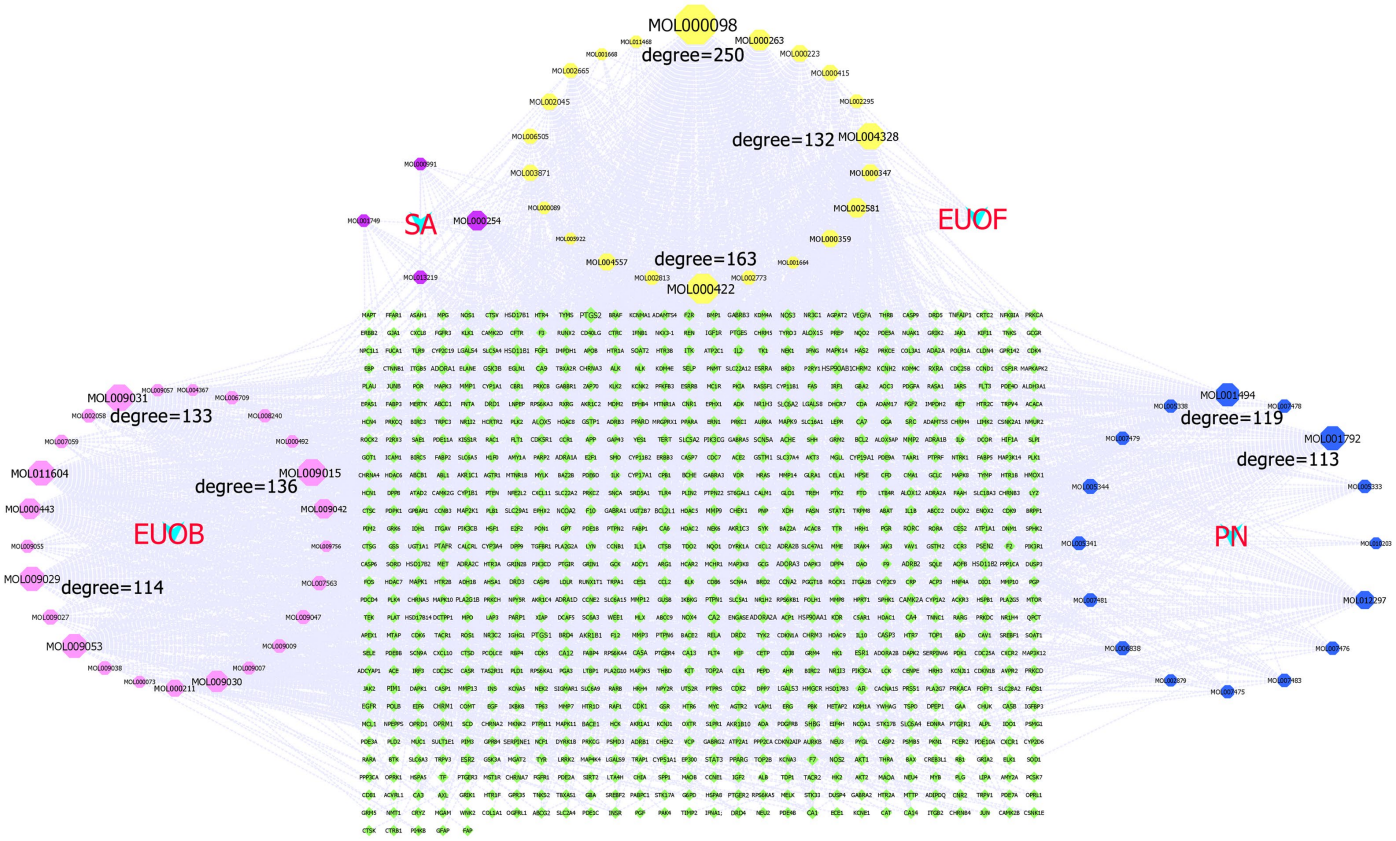
The H-A-T network diagram: the green diamond denotes the potential targets of active compounds, the four light blue “V” shapes represent the four active compounds of EUOB, EUOF, PN and SA, respectively, the pink octagon represents the active compounds of EUOB, the blue octagon represents the active compounds of PN, the purple octagon represents the active compounds of S2, while the yellow octagon represents the common compound of 4 herbs.

A total of 67 active components and 817 potential targets of EEPS were imported into the Cytoscape 3.9.1 software for construction of the H-A-T network. In Figure 4, each node represents different active components and targets, and connections between nodes represent interactions between active components and their targets. Network topology analysis revealed 888 nodes with 3,103 edges, with an average node degree of 6.597 and 189 nodes with a degree greater than the average degree. The larger the node, the larger the degree value, indicating more importance in the network. The top 8 of the 67 compounds in EEPS were MOL000098 (Quercetin, EUOB, EUOF, PN, SA), MOL000422 (Kaempferol, EUOB, EUOF, PN, SA), MOL009015 ((−)-Tabernemontanine, EUOB), MOL009031 ((9R) Cinchonan-9-al, 6′-methoxy-, (9R)-, EUOB), MOL004328 (Naringenin, EUOB, EUOF), MOL011604 (Syringetin, EUOB), MOL009029 (Dehydrodiconiferyl alcohol 4, gamma’-di-O-Beta-D-glucopyanoside_qt, EUOB), and MOL001792 (DFV, PN). Among them, quercetin was associated with 246 targets, such as prostaglandin-endoperoxide synthase 1 (PTGS1), androgen receptor (AR), peroxisome proliferator activated receptor gamma (PPARG), and prostaglandin-endoperoxide synthase 2 (PTGS2); kaempferol was associated with 159 targets, such as nitric oxide synthase 2 (NOS2), PTGS1, AR, and PPARG, followed by (−)-tabernemontanine, ((9R) Cinchonan-9-al, 6′-methoxy-, (9R)-, naringenin, syringetin, dihydrodiconiferyl alcohol 4, gamma’-di-O-Beta-D-glucopyanoside_qt and DFV), which were also associated with 135, 132, 130, 118, 113, and 112 targets, respectively.

### 3.4. ADMET study of key components

Eight key components were compiled using the ADMET screening function of the ADMETIab 2.0 database. Chemical structures of these components and predicted results are shown in Table 1. According to Lipinski’s Rule of Five (74), the molecular weight of the compound should not be greater than 500 (MW ≤ 500), the log of the octanol/water partition coefficient should not be greater than 5 (LogP ≤5), the number of hydrogen bond acceptors in the compounds should not be greater than 10 (HBA < 10), and the number of hydrogen bond donors (including hydroxyl groups, amino groups, etc.) in the structure of compounds should not be greater than 5 (HBD ≤ 5). The predicted results suggested that all the key components complied with limits of the Lipinski rule, indicating they have good bioavailability.

**Table 1:** ADMET profile of the key components.

No.	Compounds	Structure	Lipinski rules								
			MW ≤ 500	LogP ≤4.15	HBA < 10	HBD ≤ 5	LopS	BBB	Lipinski’s violations	Sascore	TPSA (Å2)
1	Quercetin	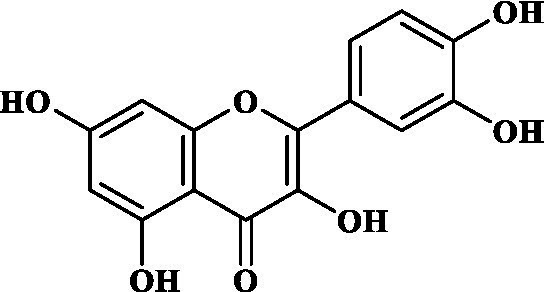	302.24	2.155	7	5	−3.671	0.008	0	2.545	131.36
2	Kaempferol	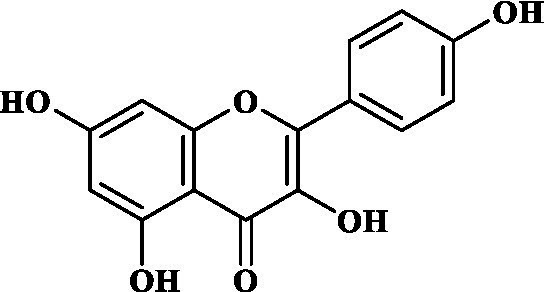	286.05	2.656	6	4	−3.624	0.009	0	2.375	111.13
3	(−)-Tabernemontanine	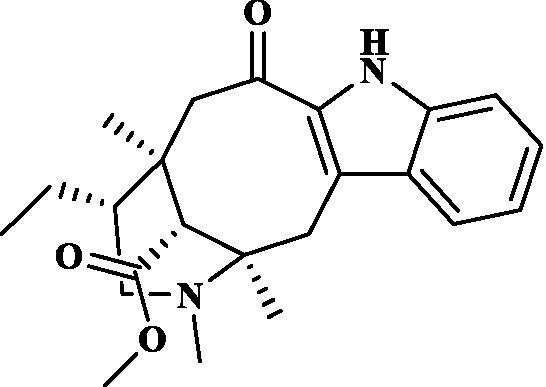	354.19	3.359	5	1	−4.145	0.974	0	4.586	62.4
4	Cinchonan-9-al, 6′-methoxy-, (9R)-	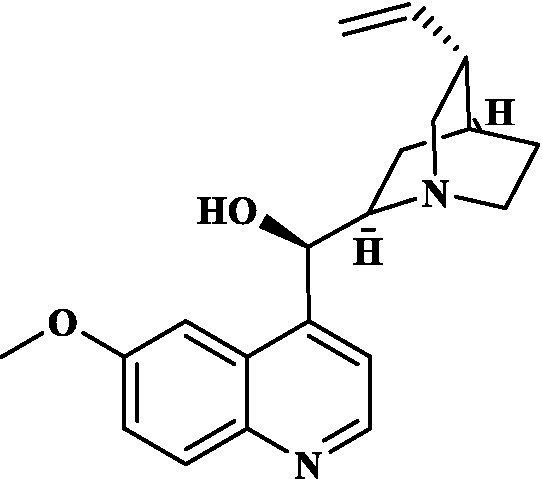	324.18	2.719	4	1	−2.751	0.868	0	4.516	45.59
5	Naringenin	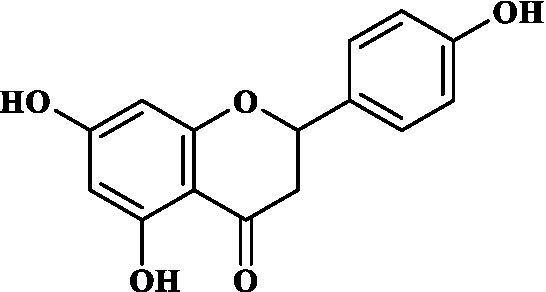	272.07	2.562	5	3	−3.876	0.042	0	2.825	86.99
6	Syringetin	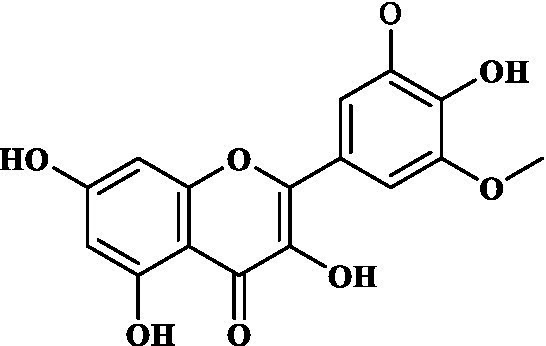	346.07	2.463	8	4	−3.817	0.004	0	2.541	129.59
7	Dehydrodiconiferyl alcohol 4,gamma’-di-O-beta-D-glucopyanoside_qt	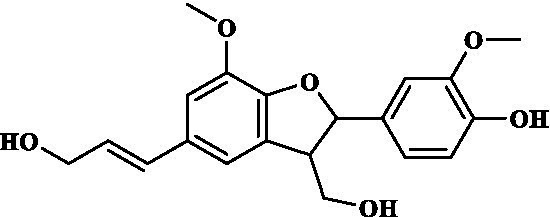	358.14	1.842	6	3	−3.487	0.438	0	3.429	88.38
8	DFV	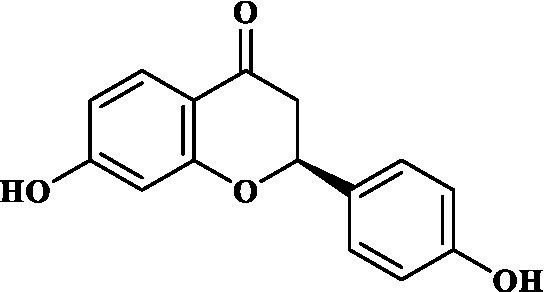	256.07	2.5	4	2	−3.892	0.078	0	2.657	66.76

### 3.5. Construction of the PPI network and key target selection

**Figure 5:**
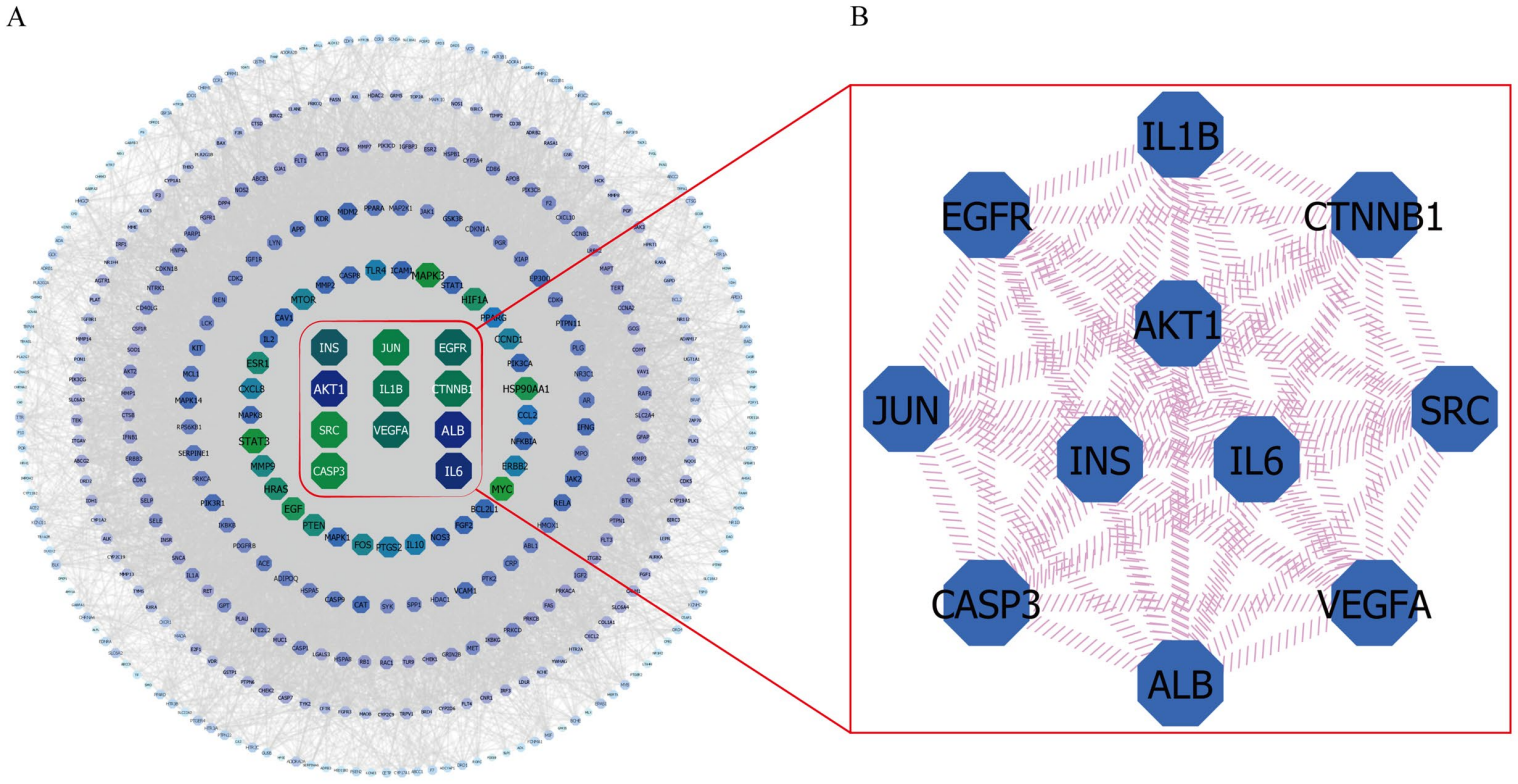
The PPI network and a key subnetwork: **(A)** Protein – protein interaction network (PPI); **(B)** Hub genes in the PPI network.

A total of 413 overlapping targets were imported into the STRING 11.5 database. After hiding the unrelated nodes, the exported data in TSV format were imported into the Cytoscape 3.9.1 software to establish the protein–protein interaction (PPI) network diagram. Analysis of network topology revealed that the network contained 411 nodes and 11,117 edges, while the average degree of nodes was 54.097. In Figure 5, proteins and protein–protein interactions were represented by network nodes and edges. The size of nodes indicates the size of the degree value of connections, where the larger the nodes and the darker the color, the larger the corresponding degree value (75). Figure 5A shows that the closer to the center of the concentric circles, the larger the nodes, and the darker the color, which also means that the closer to the center of the concentric circles, the more important the nodes. Herein, the Cytoscape 3.9.1 software was used to identify the key anti-fatigue targets of EEPS by comparing the degree values. In Figure 5B, our topology studies revealed that the nodes that ranked high in degree value, including AKT serine/threonine kinase 1 (AKT1, degree = 251), interleukin 6 (IL6, degree = 237), insulin (INS, degree = 213), epidermal growth factor receptor (EGFR, degree = 208), vascular endothelial growth factor A (VEGFA, degree = 207), catenin beta 1 (CTNNB1, degree = 201), interleukin 1 beta (IL1B, degree = 201), jun proto-oncogene (JUN, degree = 195), caspase 3 (CASP3, degree = 192), and SRC proto-oncogene (SRC, degree = 191), were key targets of EEPS for fatigue treatment.

### 3.6. Gene ontology enrichment and KEGG pathway enrichment analyses

**Figure 6:**
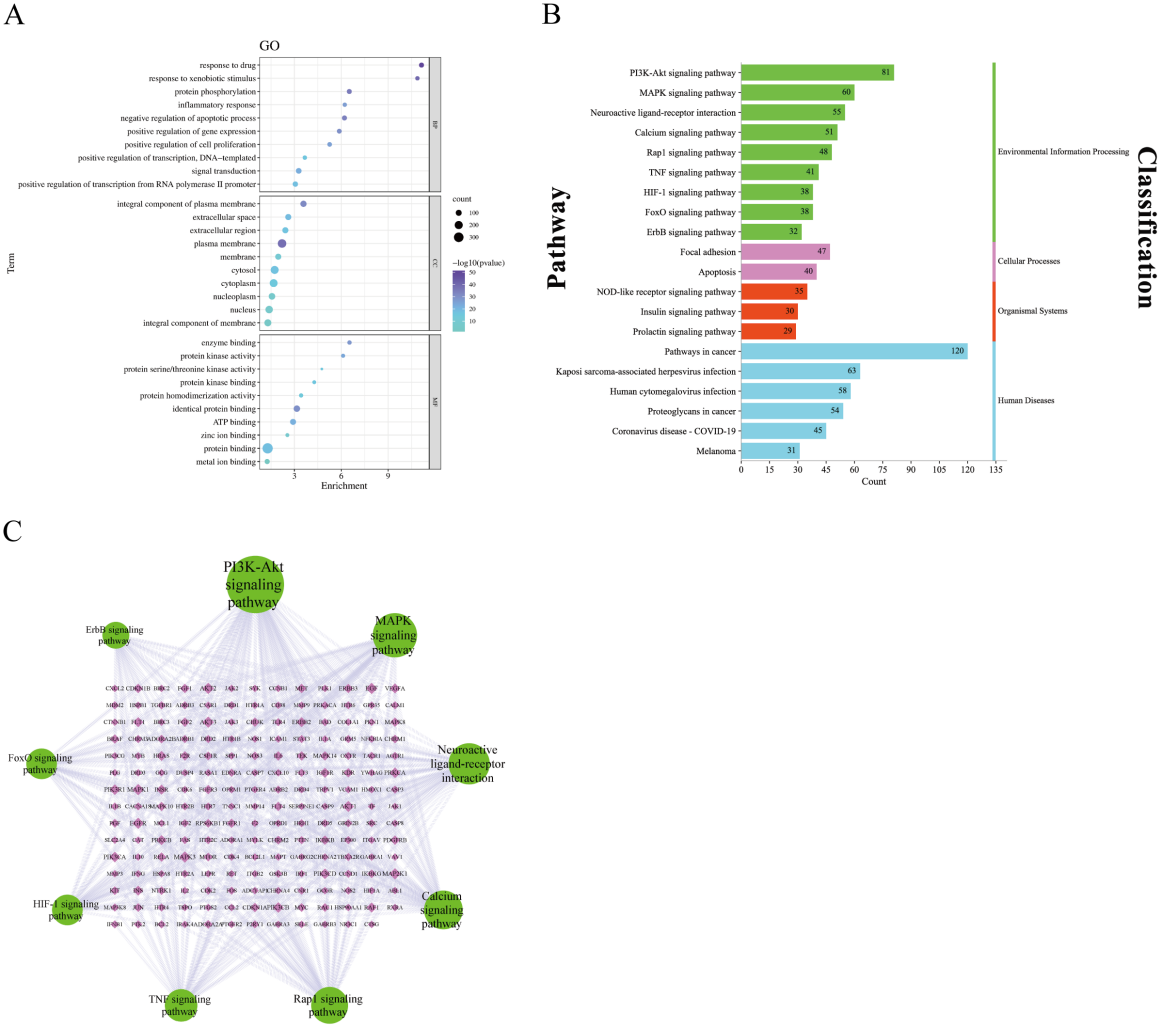
**(A)** Bubble diagram of GO enrichment analysis; **(B)** Bar diagram of KEGG pathway enrichment; **(C)** Pathway-gene network diagram: green circular nodes represent the nine signaling pathways that were mainly enriched in the environmental information processing metabolic pathway, and pink diamond nodes represent the genes enriched in each pathway.

GO enrichment and KEGG pathway analyses were performed using the DAVID v2022q2 database to investigate the biological functions of the 413 overlapping targets. A total of 1,636 GO enrichment entries were obtained; including 1,232 biological process (BP) GO entries, 145 cellular component (CC) GO entries, 259 molecular function (MF) GO entries, and 189 KEGG signaling pathways. Bubble diagram of the GO enrichment results was visualized using the bioinformatics online platform. The enrichment degree is indicated by color. The darker the color, the more significant the enrichment. In Figure 6A, the GO biological process (BP) terms were mainly enriched in signal transduction, positive regulation of transcription from RNA polymerase II promoter, response to drug, protein phosphorylation, and negative regulation of apoptotic processes among others. For CC, the major enriched GO terms were plasma membrane, cytosol, cytoplasm, and nucleus among others. In MF, genes were mainly enriched in protein binding, identical protein binding, ATP binding, and metal ion binding among others. To study the anti-fatigue mechanisms of EEPS at the pathway level, 20 fatigue-related pathways were screened and plotted in a bar chart Figure 6B. The KEGG enrichment analysis showed that nine signaling pathways (PI3K-Akt, MAPK, neuroactivator-receptor interaction, calcium, Rap1, TNF, HIF-1, FoxO and ErbB signaling pathways) were enriched in environmental information processing metabolic pathway; focal adhesion and apoptosis were enriched in the cellular process metabolic pathway; the NOD-like receptor, insulin and prolactin signaling pathways were enriched in organismal systems metabolic pathway; while pathways in cancer, Kaposi sarcoma-associated herpesvirus infection, human cytomegalovirus infection, proteoglycans in cancer, coronavirus disease—COVID-19 and melanoma were enriched in the human diseases metabolic pathway. Most of the pathways were enriched in metabolic pathway Environmental Information Processing, indicating that this metabolic pathway plays an important role in anti-fatigue processes. Nine pathways enriched in environmental information processing and the genes associated with the pathway were imported into a pathway-gene network using the Cytoscape 3.9.1 software (accessed on 2^nd^ November 2022). In Figure 6C, nodes indicate the pathways and genes enriched in these pathways, while the connecting lines between the nodes indicate the correlations between pathways and genes. A total of 203 nodes and 444 edges were found in the network, with an average degree value of connexions of 4.374. In such networks, the larger the nodes, the larger the degree. KEGG pathway analysis revealed that the top 3 pathways with higher degrees included the PI3K-Akt signaling pathway (degree = 81), MAPK signaling pathway (degree = 60), and neuroactivator-receptor interaction pathway (degree = 55), which were considered active pathways. These hub genes and related signaling pathways play important roles in the anti-fatigue process of EEPS.

### 3.7. Molecular docking

**Figure 7:**
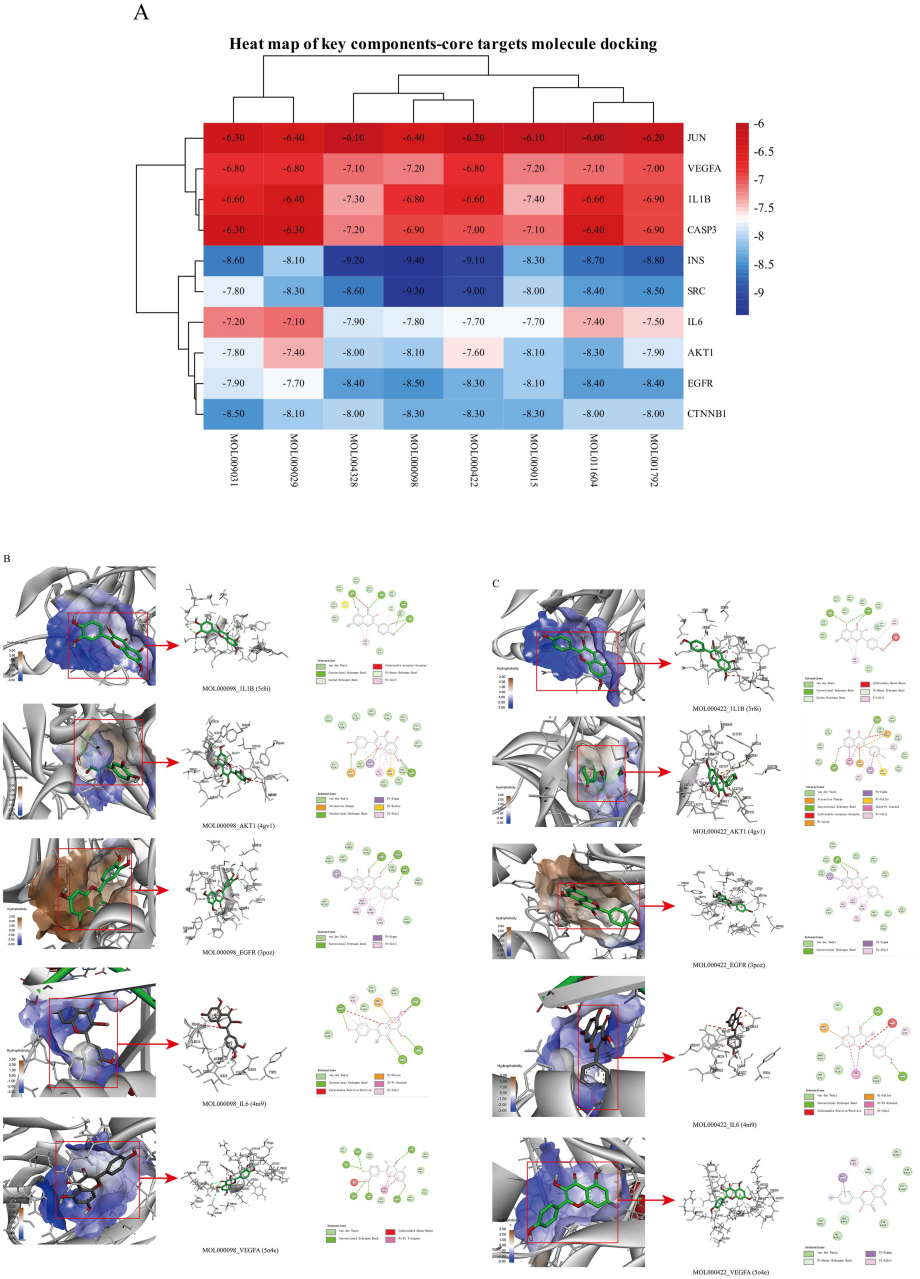
**(A)** Heat map of docking of key compounds with core targets. **(B)** 3D and 2D maps of quercetin (MOL000098) docking with AKT1, IL6, EGFR, VEGFA and 1L1B. **(C)** 3D and 2D maps of kaempferol (MOL000422) docking with AKT1, IL6, EGFR, VEGFA and 1L1B.

Molecular docking simulations were performed using the AutoDock Tool to verify the reliability of interactions between important proteins and key compounds. Crystal structures of AKT1, IL6, INS, EGFR, VEGFA, CTNNB1, IL1B, JUN CASP3 and SRC were compiled by searching against the UniProt and RCSB Protein Data Bank (PDB) databases. The chemical structures of eight key components, including MOL000098 (Quercetin), MOL000422 (Kaempferol), MOL009015 ((−)-Tabernemontanine), MOL009031 ((9R) Cinchonan-9-al, 6′-methoxy-, (9R)-), MOL004328 (Naringenin), MOL011604 (Syringetin), MOL009029 (Dehydrodiconiferyl alcohol 4, gamma’-di-O-Beta-D-glucopyanoside_qt), and MOL001792 (DFV) were collated from the PubChem database. Then, molecular docking was performed to assess the binding between the top 10 targets and the key compounds through AutoDock 4.0 software. The lower the binding energy, the more stable the binding between the target and compound, and the more likely interactions will occur. The docking results shown in Figure 7A; Supplementary Table S2 indicate that all the key compounds have good affinity for core targets. We found that active ingredients, quercetin and kaempferol, the targets (AKT1, IL6, EGFR, VEGFA and 1L1B), showed better effects in the anti-fatigue process (Figures 7B,C). The docking results between quercetin and kaempferol and 1L1B, AKT1, EGFR, IL6 and VEGFA are shown in 2D and 3D. Active components (ligands) are embedded in targets (proteins) and interconnected with residues of targets by various interaction forces (Supplementary Table S3). Root mean square deviation (RMSD) is an important indicator for assessing the reliability of interaction patterns calculated by the docking procedure and requires RMSD <2.0 Å. In Table 2, all RMSD values are less than 2.0 Å, i.e., the receptor–ligand interactions are reliable.

**Table 2:** Molecular docking parameters of quercetin, kaempferol, and 1L1B, AKT1, EGFR, IL6, and VEGFA.

Compounds (Ligand)	Targets (PDB ID)	RMSD (Å)	Binding energy (kJ/mol)	Center	Size
MOL000098 (quercetin)	1L1B (5r8i)	0	−6.8	*x* = 40.006, *y* = 8.701, *z* = 56.427	*x* = 21, *y* = 21, *z* = 21
	AKT1 (4gv1)	0	−8.1	*x* = −20.8, *y* = 7.36, *z* = 12.229	*x* = 21, *y* = 21, *z* = 21
	EGFR (3poz)	1.953	−8.5	*x* = 16.656, *y* = 32.531, *z* = 18.518	*x* = 21, *y* = 21, *z* = 33
	IL6 (4ni9)	0	−7.8	*x* = 16.922, *y* = 20.285, *z* = −0.956	*x* = 21, *y* = 21, *z* = 21
	VEGFA (5o4e)	0	−7.2	*x* = 157.73, *y* = 8.079, *z* = 144.882	*x* = 21, *y* = 21, *z* = 21
MOL000422 (kaempferol)	1L1B (5r8i)	1.71	−6.6	*x* = 4 0.006, *y* = 8.701, *z* = 56.427	*x* = 21, *y* = 21, *z* = 21
	AKT1 (4gv1)	0	−7.6	*x* = −20.8, *y* = 7.36, *z* = 12.229	*x* = 21, *y* = 21, *z* = 21
	EGFR (3poz)	0	−8.3	*x* = 16.656, *y* = 32.531, *z* = 18.518	*x* = 21, *y* = 21, *z* = 33
	IL6 (4ni9)	1.44	−7.7	*x* = 16.922, *y* = 20.285, *z* = −0.956	*x* = 21, *y* = 21, *z* = 21
	VEGFA (5o4e)	0	−6.8	*x* = 157.73, *y* = 8.079, *z* = 144.882	*x* = 28, *y* = 21, *z* = 21

## 4. Discussion

Fatigue is a long protracted chronic disease that is cannot be easily cured within a short time (76). Currently, multiple mechanisms for fatigue have been proposed, including the free radical hypothesis, metabolite accumulation hypothesis, stress response system hypothesis, among others. Due to the complex pathogenesis of fatigue, a novel effective therapeutic strategy should be urgently developed. Given that fatigued patients often require long-term treatment, the adverse effects associated with long-term administration of drugs cannot be ignored (77). Hence, functional foods have become an effective alternative. “Homology of medicine and food” is a well-accepted TCM theory, which implies that some herbal medicines share the same origin with food (78). On this basis, we concluded that these TCMt can also be used as raw materials to produce functional foods due to their well-defined efficacy and excellent safety. According to the “Qi and Blood Theory” and the related documents from the National Health Commission of the People’s Republic of China, we selected four kinds of “Qi-tonifying medicine” herbal medicines, EUOB, EUOF, PN and SA, to prepare a herbal medicine compound called EEPS. This compound can potentially to serve as an anti-fatigue functional food. Although functional foods have been shown to improve the health of the human body, there are some issues that need to be solved. For instance, the mechanisms of actions for many functional foods are not well-defined, especially the species derived from natural products. Recently, network pharmacology has been shown to be a reliable and efficient tool for exploring the complex mechanisms and multiple effects of TCM.

In this study, Network pharmacology and molecular docking strategies were employed to investigate the complex anti-fatigue mechanisms of EEPS. A total of 67 key compounds with anti-fatigue effects were identified through analysis of literature and researching several databases. After collecting and collating their potential anti-fatigue targets, we constructed a network diagram of ‘herbals-compounds-targets’. The analysis revealed that most of the candidate compounds of EEPS affected multiple fatigue-related targets. Subsequently, 8 key active compounds were selected based on their degree values. Next, a ADMETLab 2.0 database was used to predict drug-like properties of these compounds. The results showed that all these key active compounds satisfied the principles of Lipinski’s ‘rule of five’, which means that they can be easily absorbed by humans when taken orally. We then analyzed the top 8 key active compounds based on their chemical structure. It was observed that the compounds had various chemical structures including flavonoids, lignans, alkaloids, and phenolic acids. The top-ranked active compounds were quercetin and kaempferol, both of which are flavonoids. Although they were found in both EUOB and EUOF, the levels of flavonoids in EUOF were higher than in EUOB (43). In addition, they were the main components of flavonoids in SA (79). As detailed in previous literature, 3-week-old BALB/c mice were fed on 0.005% quercetin for 6 weeks, and then subjected to the weight-loaded swimming test (80). Experimental results demonstrated that quercetin can improve resistance capacity to fatigue by maintaining antioxidant capacity, restoring muscle glycogen stores, and improving muscle function. In a previous randomized, double-blind, placebo-controlled, crossover design study on student volunteers by Liu (81), it was found that the intake of quercetin altered MDA levels in participants and inhibited the disruption to lipid peroxidation, thereby postponing the occurrence of fatigue. Meanwhile, quercetin has been shown to exert its anti-fatigue capacity by improving the antioxidant capacity by increasing the activity of SOD and GSH-Px. These results suggested that quercetin may be a promising anti-fatigue compound. Kaempferol is another critical flavonoid that is found in EUOB, EUOF and SA. This compound is known to possess multiple biological activities, such as antioxidant and anti-inflammatory properties (82). Liu et al., reported that kaempferol not only protected beta cells against dRib-induced oxidative damage but also inhibited intracellular ROS, apoptosis, and lipid peroxidation (81). The *in vitro* results indicated that kaempferol and quercetin alone and in combination can induce antioxidant response elements (AREs) and increase Nrf2 protein levels, thereby exert antioxidant activity. It is worth mentioning that the free radical scavenging activities of kaempferol were enhanced when used in combination with quercetin (83). According to the oxidative hypothesis, increasing the activity of antioxidative enzymes and alleviating oxidative stress can effectively alleviate fatigue. We further note that *Panax notoginseng* saponins (PNS) are widely considered to be the main anti-fatigue active compound of PN (47). With in-depth studies, some flavonoids, including quercetin and kaempferol, have also been identified in PN. A study based on animal experiments showed that combining PNS with other notoginseng flavones increased its biological activity (42). This work provided valuable experimental evidence and clues for the design of anti-fatiguing compounds. Syringaresinol and dehydrodiconiferyl alcohol 4, gamma’-di-O-Beta-D-glucopyanoside_qt, belong to the lignan class of compounds. In Chinese pharmacopeia (2020), the content of lignans is considered the quality standard for EUOB. Although few studies have explored their anti-fatigue activity, there is little information to guide further investigations. For example, *in vitro* results showed that syringaresinol exerted antioxidant activities, hence could affect the pathogenesis of fatigue (84). Besides, syringaresinol can activate SIRT1, which increases the antioxidant capacity through activation of nuclear factor (erythroid-derived 2)-like 2 (85). In general, compounds with similar chemical structures tend to have similar biological properties. According to this principle, studies on the anti-fatigue activity of other lignans from TCM were performed. Jin et al. (86) found that after administration of a lignan-rich extract for 30 days increased the swimming ability of male Kunming mice significantly. Further studies showed that both blood urea nitrogen (BUN) and BLA were increased, whereas the GSH levels and activities of antioxidant enzymes were decreased. Additionally, based on data mining, two alkaloids isolated from EUOB might were found to exert anti-fatigue effects of EEPS. As discussed above, the top 8 key compounds extracted from different herbs possess diverse chemical structures and these compounds may exert anti-fatigue action via multiple different mechanisms. Besides, synergy against fatigue was achieved when some of the key bioactive compounds were properly combined in a hybrid approach. Additionally, we also note that EUOB and EUOF contain the largest number of key compounds, suggesting that these two herbs could be used as the major ingredients in EEPS.

In our further studies, PPI networks of the EEPS targets and fatigue-related targets were constructed to identify 413 potential targets of EEPS in the treatment of fatigue. Subsequently, network pharmacology analyzes were conducted. The Analysis Network function in Cytoscape 3.9.1 was applied to identify the top 10 core targets, including AKT, 1IL6, IL1B, EGFR, and VGEFR. Coincidentally, the key compounds mentioned in the preceding paragraph were found to act on multiple core targets via multiple pathways. AKT1 showed the highest degree of node, and has been demonstrated to regulate many biological processes, including cellular growth, apoptosis, survival, and angiogenesis (87–89). It is generally accepted that oxidative stress (free radicals) leads to mitochondrial dysfunction and cell damage (90), which in turn results in fatigue. Numerous studies have proven that mitochondria are particularly susceptible to oxidative stress, and mitochondrial oxidative stress in turn promotes oxidative stress-induced damage to the cells (91, 92). According to the report by Afolayan AJ, AKT1 may be a key regulator of mitochondrial oxidative stress and vascular function (93). Animal experiments have demonstrated that activation of AKT1 can protect cells against oxidative stress and it may play be an effective treatment for fatigue. In the O’s study, 50 or 100 mg/(kg·d) ginseng saponin fraction and its metabolic products were administered orally to male ICR mice once a day for 5 days. This caused significant improvement on the weight-loaded forced swimming ability (94). Meanwhile, the treatments decreased the levels of corticosterone, lactate, and creatinine. This study demonstrated that the ginseng saponin fraction may possess anti-fatigue effects. Alternatively, in a study conducted by Adachi et al., SPFACR mice were used to explore the mechanism and potential targets of ginseng. They found that AKT1 levels in muscle tissue were increased following oral administration of ginseng saponin. These experiments demonstrated that the regulation of AKT1 by herbal medicine can improve the anti-fatigue capacity of the organism. Among these core targets, EGFR has also attracted our attention. Activation of EGFR and its downstream PI3K-AKT–mTOR signaling pathway can regulate cell proliferation and survival (95, 96). A previous study by Kim et al. demonstrated that EGFR knockdown not only affected ROS scavenging function but also damaged mitochondrial structures and increased the vulnerability of these cells to oxidative stress (97). These findings suggest that targeting AKT1 and EGFR to attenuate oxidative stress to improve anti-fatigue capacity effects.

A review of literature showed a strong relationship between oxidative stress and inflammation (98–101). Furthermore, numerous preclinical and clinical studies have shown a close association between inflammation and fatigue (102). Given the crucial role of inflammation in patients with fatigue, several published studies have demonstrated the notion that anti-fatigue effects can be achieved by regulating inflammatory responses. Among the core inflammatory factors targets, both IL1B and IL6 are well recognized as key proinflammatory cytokines with a significant proinflammatory effect (103). The IL-6 levels in the serum of fatigued rats were observed to be increased (104). A similar phenomenon was reported in chronic fatigue patients (105). A study of cancer patients showed that fatigue severity was significantly associated with IL-6 levels (106). Considering that regulation of IL-6 expression can relieve inflammation, we believe that IL-6 may be a promising target for the treatment of fatigue. VEGFA belongs to the VEGF growth factor family and is believed to promote multiple differentiation capabilities of angiogenesis, including enhancement of vascular permeability and promotion of effects of vascular permeability (107). Recently, VEGFA was reported to play an important role in inflammation. For example, Wang et al. demonstrated that VEGF was most closely related to brain inflammation during the COVID-19 outbreak and therefore could be a potential therapeutic target for SARS-CoV-2 patients with neurological symptoms (108). In another clinical study of 28 patients with tuberculosis (Tb), it was found that the levels of VEGFA were an important determinant of DS-TB patients’ clinical status (109). They proposed that VEGFA was the main factor contributing to inflammation and angiogenesis. Moreover, Jin et al. found that overexpression of VEGFA in patients with peritoneal dialysis (PD) was positively related to the degree of peritoneal inflammation (110). Furthermore, an *in vivo* study demonstrated that VEGF blockers could exert anti-inflammatory effects in CIA rat models (111). Inhibition of VEGFA through pharmacological agents or pharmacological treatment effectively controlled granulomatous inflammation.

Subsequently, KEGG and GO pathway enrichment analyses were performed to identify the biological functions of EEPS. The results of the analysis suggested that EEPS can exert anti-fatigue effects through multiple signaling pathways, including the PI3K-Akt signaling pathway, the MAPK signaling pathway, and the Rap1 signaling pathway. The top KEGG pathway was the PI3K-Akt signaling pathway. We further conducted a literature search on the PubMed using a combination of the words ‘PI3K/Akt’, ‘*Eucommia ulmoides* Oliver’, ‘*Eugenia caryophyllata*’ and *‘Notoginseng Radix et Rhizoma*’. Previous studies have shown that these herbs, their extracts or their formulations are affect the PI3K/Akt pathways (112–114). This further confirms the anti-fatigue potential of this TCM compound. Another key pathway is the MAPK signaling pathway. This pathway has been widely acknowledged to be a crucial target for anti-inflammatory therapy in many studies. *In vitro* and *in vivo* assays have shown that multiple compounds in EEPS can suppress inflammation by regulating the MARK signaling pathways (115–117). In addition, molecular docking studies showed that the key active compounds had strong binding affinity to these core anti-fatigue target proteins. Collectively, these results confirmed the results of the constructed network pharmacology.

This systematic study found that alkaloids, flavonols, and other active compounds of EEPS can exert anti-fatigue effects through multiple targets and multiple signaling pathways. Among these, the antioxidant and anti-inflammatory pathways are two of the most important molecular pathways. Overall, our results reveal the actual value of network pharmacology tools in mechanistic research on functional foods. Besides, our findings contribute to the development of the functional foods industry in several ways. Firstly, functional foods can reduce consumers’ concerns and increase sales for the product. Secondly, for functional food manufacturers, identifying the key active ingredients can improve the product quality control. Additionally, investigation of potential molecular mechanisms increases the awareness of the pathomechanism of fatigue, which will help to identify new potential targets for development of disease therapies. However, further animal experimental verification is needed to validate the value of this conclusion.

## 5. Conclusion

This study investigated anti-fatigue effects of functional foods based on the traditional medicine theories and the relevant guidance document published by the Chinese Ministry of Health. In addition, four safe herbs suitable for food ingredients were chosen to prepare anti-fatigue formulations. Subsequently, network pharmacology and molecular docking were performed to identify the potential pharmacodynamic substances, the core targets, and the key signaling pathways. This study provides scientific proof of the anti-fatigue effects of EEPS. In addition, based on the findings of this investigation, we suggest that biologically safe herbs can be used as active ingredients for the formulation of functional foods.

## Data availability statement

The datasets presented in this study can be found in online repositories. The names of the repository/repositories and accession number(s) can be found in the article/Supplementary material.

## Author contributions

YW, WH, and YH: conceptualization. YW and WH: funding acquisition. YW, YM, WH, and YH: investigation. YW and YM: methodology and writing—original draft. YM: software. YM, JC, and YH: supervision. YW, YM, RX, and FC: validation. YW, YM, and YH: writing—review and editing. YW and YM: contributed equally to this work. All authors contributed to the article and approved the submitted version.

## Funding

The authors greatly appreciate the support for this research from National Science Foundation of China (Grant nos. 21602090 and 82160292) and the Open Project of Key Laboratory of Prevention and treatment of cardiovascular and cerebrovascular diseases Ministry of Education (Grant nos. XN202030 and XN201922).

## Conflict of interest

The authors declare that the research was conducted in the absence of any commercial or financial relationships that could be construed as a potential conflict of interest.

## Publisher’s note

All claims expressed in this article are solely those of the authors and do not necessarily represent those of their affiliated organizations, or those of the publisher, the editors and the reviewers. Any product that may be evaluated in this article, or claim that may be made by its manufacturer, is not guaranteed or endorsed by the publisher.

## Abbreviations

TCM: Traditional Chinese medicine
EUOB: *Eucommia ulmoides* Oliver bark
EUOF: *Eucommia ulmoides* Oliver male flower
PN: *Panax notoginseng*
SA: *Syzygium aromaticum*
EUO: *Eucommia ulmoides* Oliver
EEPS: EUOB, EUOF, PN and SA
PNS: *Panax notoginseng* saponins
TCMSP: Traditional Chinese Medicine Systems Pharmacology Database and Analysis Platform
OB: Oral bioavailability
DL: Drug likeness
OMIM: Online Mendelian Inheritance in Man
TTD: Therapeutic Target Database
H - A – T: Herbal medicines—Active compounds—Targets
ADMET: Absorption, distribution, metabolism, excretion, toxicity
MW: Molecular weight
LogP: Log of the octanol/water partition coefficient
HBA: Hydrogen bond acceptors
HBD: Hydrogen bond donors
LopS: Log of the aqueous solubility
BBB: Blood – Brain Barrier
TPSA: Topological Polar Surface Area
Sascore: Synthetic accessibility scoreblood - brain barrier
PPI: Protein - protein interaction
GO: Gene Ontology
BP: Biological process
CC: Cellular component
MF: Molecular function
KEGG: Kyoto Encyclopedia of Genes and Genomes
PTGS1: Prostaglandin-endoperoxide synthase 1
AR: Androgen receptor
PPARG: Peroxisome proliferator activated receptor gamma
PTGS2: Prostaglandin-endoperoxide synthase 2
NOS2: Nitric oxide synthase 2
AKT1: AKT serine/threonine kinase 1
IL6: Interleukin 6
INS: Insulin
EGFR: Epidermal growth factor receptor
VEGFA: Vascular endothelial growth factor A
CTNNB1: Catenin beta 1
IL1B: Interleukin 1 beta
JUN: Jun proto-oncogene
CASP3: Caspase 3
SRC: SRC proto-oncogene

## References

[1] Azzolino D Arosio B Marzetti E Calvani R Cesari M. Nutritional status as a mediator of fatigue and its underlying mechanisms in older people. Nutrients. (2020) 12:444. doi: 10.3390/nu12020444, PMID: 32050677 PMC7071235

[2] Zielinski MR Systrom DM Rose NR. Fatigue, sleep, and autoimmune and related disorders. Front Immunol. (1827) 10:10. doi: 10.3389/fimmu.2019.01827, PMID: 31447842 PMC6691096

[3] Davis JM Bailey SP. Possible mechanisms of central nervous system fatigue during exercise. Med Sci Sports Exerc. (1997) 29:45–57. doi: 10.1097/00005768-199701000-00008 9000155

[4] Yang D Lian J Wang L Liu X Wang Y Zhao X. The anti-fatigue and anti-anoxia effects of Tremella extract. Saudi J Biol Sci. (2019) 26:2052–6. doi: 10.1016/j.sjbs.2019.08.014, PMID: 31889793 PMC6923490

[5] Yang X Li F Liu Y Li D Li J. Study on the correlation between NF-κB and central fatigue. J Mol Neurosci. (2021) 71:1975–86. doi: 10.1007/s12031-021-01803-z, PMID: 33586033 PMC8500872

[6] Mehandru S Merad M. Pathological sequelae of long-haul COVID. Nat Immunol. (2022) 23:194–202. doi: 10.1038/s41590-021-01104-y, PMID: 35105985 PMC9127978

[7] Raveendran AV Jayadevan R Sashidharan S. Long COVID: An overview. Diabetes Metab Syndr. (2021) 15:869–75. doi: 10.1016/j.dsx.2021.04.007, PMID: 33892403 PMC8056514

[8] Lopez-Leon S. Wegman-Ostrosky T. Perelman C. Sepulveda R. Rebolledo P. A. Cuapio A.. (2021). More than 50 long-term effects of COVID-19: a systematic review and meta-analysis. med Rxiv [Preprint]. doi: 10.1101/2021.01.27.21250617. PMC8352980 34373540

[9] Ma X Chen H Cao L Zhao S Zhao C Yin S. Mechanisms of physical fatigue and its applications in nutritional interventions. J Agric Food Chem. (2021) 69:6755–68. doi: 10.1021/acs.jafc.1c01251, PMID: 34124894

[10] Ross S. Functional foods: the Food and Drug Administration perspective. Am J Clin Nutr. (2000) 71:1735S–8S. doi: 10.1093/ajcn/71.6.1735S 10837331

[11] Siró I Kápolna E Kápolna B Lugasi A Functional food. Product development, marketing and consumer acceptance—a review. Appetite. (2008) 51:456–67. doi: 10.1016/j.appet.2008.05.060, PMID: 18582508

[12] Tatullo M Marrelli B Benincasa C Aiello E Amantea M Gentile S. Potential impact of functional biomolecules-enriched foods on human health: a randomized controlled clinical trial. Int J Med Sci. (2022) 19:563–71. doi: 10.7150/ijms.70435, PMID: 35370460 PMC8964315

[13] Shi X Chang M Zhao M Shi Y Zhang Y. Traditional Chinese medicine compounds ameliorating glomerular diseases via autophagy: a mechanism review. Biomed Pharmacother. (2022) 156:113916. doi: 10.1016/j.biopha.2022.113916, PMID: 36411609

[14] Shang H Zhang K Guan Z Zhang X. Optimization of evidence-based research in the prevention and treatment of coronary heart disease with traditional Chinese medicine: a comprehensive review. J Tradit Chin Med Sci. (2022) 9:100–7. doi: 10.1016/j.jtcms.2022.04.004

[15] Wang J Zheng M Guo Q Lan H Wu S Zhang J. Dominant diseases of traditional Chinese medicine (TCM). Integr Med Res. (2022) 11:100872. doi: 10.1016/j.imr.2022.100872, PMID: 35855936 PMC9287630

[16] Narayanankutty A Kunnath K Famurewa AC Ramesh V Rajagopal R Alfarhan A. Variations in the composition, cytoprotective and anti-inflammatory effects of natural polyphenols of edible oils extracted from fresh and dried coconut testa. Physiol Mol Plant Pathol. (2022) 117:101742. doi: 10.1016/j.pmpp.2021.101742

[17] Malayil D House NC Puthenparambil D Job JT Narayanankutty A. *Borassus flabellifer* haustorium extract prevents pro-oxidant mediated cell death and LPS-induced inflammation. Drug Chem Toxicol. (2022) 45:1716–22. doi: 10.1080/01480545.2020.1858854, PMID: 33307839

[18] Famurewa AC Maduagwuna EK Folawiyo AM Besong EE Eteudo AN Famurewa OA. Antioxidant, anti-inflammatory, and antiapoptotic effects of virgin coconut oil against antibiotic drug gentamicin-induced nephrotoxicity via the suppression of oxidative stress and modulation of iNOS/NF-ĸB/caspase-3 signaling pathway in Wistar rats. J Food Biochem. (2020) 44:e13100. doi: 10.1111/jfbc.13100, PMID: 31721240

[19] Leong PK Wong HS Chen J Ko KM. Yang/Qi invigoration: an herbal therapy for chronic fatigue syndrome with yang deficiency? Evid Based Complement Alternat Med. (2015) 2015:945901. doi: 10.1155/2015/945901, PMID: 25763095 PMC4339790

[20] Panossian AG Efferth T Shikov AN Pozharitskaya ON Kuchta K Mukherjee PK. Evolution of the adaptogenic concept from traditional use to medical systems: pharmacology of stress-and aging-related diseases. Med Res Rev. (2021) 41:630–703. doi: 10.1002/med.21743 33103257 PMC7756641

[21] National Health Commission of the People’s Republic of China (2002). Available at: http://www.nhc.gov.cn/wjw/gfxwj/201304/e33435ce0d894051b15490aa3219cdc4.shtml. 10.46234/ccdcw2020.082 PMC8392946 34594648

[22] National Health Commission of the People’s Republic of China (2010). Available at: http://www.nhc.gov.cn/sps/s7891/201003/38b63b4b6f39480bba4154af127e77bb.shtml (Accessed March 25, 2023).

[23] National Health Commission of the People’s Republic of China (2014). Available at: http://www.nhc.gov.cn/sps/s7890/201405/367ce408981e4807809e107417b3d361.shtml (Accessed March 25, 2023).

[24] National Health Commission of the People’s Republic of China. (2019). Available online: http://www.nhc.gov.cn/sps/s7885/202001/b941b6138e93414cb08aed926ca3c631.shtml (Accessed March 25, 2023).

[25] National Health Commission of the People’s Republic of China (2014). Available at: http://www.nhc.gov.cn/sps/s7890/201405/367ce408981e4807809e107417b3d361.shtml (Accessed March 25, 2023).

[26] National Health Commission of the People’s Republic of China (2014). Available at: http://www.nhc.gov.cn/sps/s7890/201406/8268613682e44b1cb2098e0b9c9143d7.shtml (Accessed March 25, 2023).

[27] National Health Commission of the People’s Republic of China (2014). Available online: http://www.nhc.gov.cn/sps/s7890/201407/828404f8863a4c2392b31752a20cbe88.shtml (Accessed March 25, 2023).

[28] National Health Commission of the People’s Republic of China (2019). Available online: http://www.nhc.gov.cn/sps/s7885/202001/b941b6138e93414cb08aed926ca3c631.shtml (Accessed March 25, 2023).

[29] Wang X-J Xie Q Liu Y Jiang S Li W Li B. Panax japonicus and chikusetsusaponins: a review of diverse biological activities and pharmacology mechanism. Chin Herb Med. (2021) 13:64–77. doi: 10.1016/j.chmed.2020.12.003, PMID: 36117758 PMC9476776

[30] Fang M Meng Y Du Z Guo M Jiang Y Tu P. The synergistic mechanism of Total Saponins and flavonoids in Notoginseng&ndash;safflower against myocardial infarction using a comprehensive metabolomics strategy. Molecules. (2022) 27:8860. doi: 10.3390/molecules27248860, PMID: 36557992 PMC9782856

[31] Guo WQ Chen YG Shi RZ He K Wang JF Shao JH. 20(S)-Protopanaxdiol suppresses the abnormal granule-monocyte differentiation of hematopoietic stem cells in 4T1 breast Cancer-bearing mouse. Evid Based Complement Alternat Med. (2020) 2020:8747023–11. doi: 10.1155/2020/8747023, PMID: 32015754 PMC6982358

[32] Peng C Sang S Shen X Zhang W Yan J Chen P. In vitro anti-*Helicobacter pylori* activity of Syzygium aromaticum and the preliminary mechanism of action. J Ethnopharmacol. (2022) 288:114995. doi: 10.1016/j.jep.2022.114995, PMID: 35032584

[33] Sharma N Kar A Panda S Yadav D. Co-administration of *Pterocarpus marsupium* extract and Glibenclamide exhibits better effects in regulating hyperglycemia and associated changes in Alloxan-induced diabetic mice. Curr Top Med Chem. (2022) 22:2617–28. doi: 10.2174/1568026623666221108125036, PMID: 36366849

[34] Rauf A Akram M Anwar H Daniyal M Munir N Bawazeer S. Therapeutic potential of herbal medicine for the management of hyperlipidemia: latest updates. Environ Sci Pollut Res Int. (2022) 29:40281–301. doi: 10.1007/s11356-022-19733-7, PMID: 35320475

[35] De G Chen A Zhao Q Xie R Wang C Li M. A multi-herb-combined remedy to overcome hyper-inflammatory response by reprogramming transcription factor profile and shaping monocyte subsets. Pharmacol Res. (2021) 169:105617. doi: 10.1016/j.phrs.2021.105617, PMID: 33872811

[36] Pei B. F. (2013). Traditional Chinese medicine composition for tonifying qi and nourishing blood.

[37] Li FW Wang FY Zhang LP. Analysis on the development status and development trend of *Eucommia ulmoides* health food from the perspective of social network. Food Ind. (2022) 43:5.

[38] Chen L. (2002). A method for the preparation of clove eucommia health wine. CN1373196A[P].

[39] Liu SY Xu HJ. (2017). A health wine and its preparation method. CN107468908A[P].

[40] Shi CZ. (2013). A health wine and its preparation method. CN103451078A[P].

[41] Huang L Lyu Q Zheng W Yang Q Cao G. Traditional application and modern pharmacological research of *Eucommia ulmoides* Oliv. Chin Med. (2021) 16:73. doi: 10.1186/s13020-021-00482-7, PMID: 34362420 PMC8349065

[42] Zhao Y Tan DC Peng B Yang L Zhang SY Shi RP. Neuroendocrine-immune regulatory network of *Eucommia ulmoides* Oliver. Molecules. (2022) 27:27. doi: 10.3390/molecules27123697, PMID: 35744822 PMC9229650

[43] Peng MF Tian S Song YG Li CX Miao MS Ren Z. Effects of total flavonoids from *Eucommia ulmoides* Oliv. Leaves on polycystic ovary syndrome with insulin resistance model rats induced by letrozole combined with a high-fat diet. J Ethnopharmacol. (2021) 273:113947. doi: 10.1016/j.jep.2021.113947, PMID: 33617969

[44] Wei Y Wen X Weiye LI Huazhong YU. Research Progress of Cell Wall breaking technology and its application in *Eucommia ulmoides* male flower tea. Med Plant. (2018) 9:58–60. doi: 10.3969/j.issn.1001-3601.2018.10.028

[45] Yan YZH Zou LS Liu XH Chai C Wang SN Hua YJ. Analysis of chemical constituents in male flowers of *Eucommia ulmoides* by liquid chromatography coupled with electrospray ionization-triple quadrupole—time of flight-tandem mass spectrometry (LC—ESI—triple TOF-MS/MS). Food. Science. (2018) 39:215–21. doi: 10.7506/spkx1002-6630-201806034

[46] Ji C Zhang Q Shi R Li J Wang X Wu Z. Determination of the authenticity and origin of Panax Notoginseng: a review. J AOAC Int. (2022) 105:1708–18. doi: 10.1093/jaoacint/qsac081, PMID: 35894651

[47] Yong-xin X Jian-jun Z. Evaluation of anti-fatigue activity of total saponins of Radix notoginseng. Indian J Med Res. (2013) 137:151–5. doi: 10.1051/jp4:2004118040 PMID: 23481065 PMC3657880

[48] Lin ZXLFX Zhao YN Zhang HL Guo YS Zhang DP Zhang Q. Anti-fatigue analysis of common mechanisms of interaction of ginseng Radix et Rhizoma “Tonifying Qi” and Notoginseng Radix et Rhizoma “enriching blood”. Chin J Exp Tradit Med Formulae. (2020) 26:81–9. doi: 10.13422/j.cnki.syfjx.20201540

[49] Batiha GE Alkazmi LM Wasef LG Beshbishy AM Nadwa EH Rashwan EK. Syzygium aromaticum L. (Myrtaceae): traditional uses, bioactive chemical constituents. Pharmacol Toxicol Activ Biomol. (2020) 10:202. doi: 10.3390/biom10020202, PMID: 32019140 PMC7072209

[50] Haro-González JN Castillo-Herrera GA Martínez-Velázquez M Espinosa-Andrews H. Clove essential oil (*Syzygium aromaticum* L. Myrtaceae): extraction, chemical composition, food applications, and essential bioactivity for human health. Molecules. (2021) 26:6387. doi: 10.3390/molecules26216387 34770801 PMC8588428

[51] Li S Zhang B Jiang D Wei Y Zhang N. Herb network construction and co-module analysis for uncovering the combination rule of traditional Chinese herbal formulae. BMC Bioinformatics. (2010) 11:Article number: S6. doi: 10.1186/1471-2105-11-s11-s6, PMID: 21172056 PMC3024874

[52] Zeng P Wang XM Ye CY Su HF Tian Q. The Main alkaloids in *Uncaria rhynchophylla* and their anti-Alzheimer’s disease mechanism determined by a network pharmacology approach. Int J Mol Sci. (2021) 22:3612. doi: 10.3390/ijms22073612, PMID: 33807157 PMC8036964

[53] Wang S Ma Y Huang Y Hu Y Huang Y Wu Y. Potential bioactive compounds and mechanisms of Fibraurea recisa Pierre for the treatment of Alzheimer’s disease analyzed by network pharmacology and molecular docking prediction. Front Aging Neurosci. (2022) 14:1052249. doi: 10.3389/fnagi.2022.1052249 36570530 PMC9772884

[54] Yuriev E Agostino M Ramsland PA. Challenges and advances in computational docking: 2009 in review. J Mol Recognit. (2011) 24:149–64. doi: 10.1002/jmr.1077, PMID: 21360606

[55] Yuriev E Holien J Ramsland PA. Improvements, trends, and new ideas in molecular docking: 2012-2013 in review. J Mol Recognit. (2015) 28:581–604. doi: 10.1002/jmr.2471, PMID: 25808539

[56] Ru J Li P Wang J Zhou W Li B Huang C. TCMSP: a database of systems pharmacology for drug discovery from herbal medicines. J Cheminform. (2014) 6:13. doi: 10.1186/1758-2946-6-13, PMID: 24735618 PMC4001360

[57] Shi HY Wang WH Wang JH Xian J Yang X Li XQ. Research analysis of the current status of application of drug-like concepts in new drug development. Chin Sci Technol J (full-text version) Med Health. (2017) 1:00289.

[58] Yuan CSCW. Yner gistic car diovascular pharmacological effects and mechanism of rosea flavonoids and volatile oil based on TCMSP methods. J Shihezi Univ Nat Sci. (2016) 34:8. doi: 10.13880/j.cnki.65-1174/n.2016.06.013

[59] UniProt Consortium. UniProt: the universal protein knowledgebase in 2021. Nucleic Acids Res. (2021) 49:D480–9. doi: 10.1093/nar/gkaa1100, PMID: 33237286 PMC7778908

[60] Daina A Michielin O Zoete V. Swiss target prediction: updated data and new features for efficient prediction of protein targets of small molecules. Nucleic Acids Res. (2019) 47:W357–w364. doi: 10.1093/nar/gkz382, PMID: 31106366 PMC6602486

[61] Amberger JS Hamosh A. Searching online Mendelian inheritance in man (OMIM): a knowledgebase of human genes and genetic phenotypes. Curr Protoc Bioinformatics. (2017) 58:1.2.1-1.2.12. doi: 10.1002/cpbi.27, PMID: 28654725 PMC5662200

[62] Zhou Y Zhang Y Lian X Li F Wang C Zhu F. Therapeutic target database update 2022: facilitating drug discovery with enriched comparative data of targeted agents. Nucleic Acids Res. (2022) 50:D1398–d1407. doi: 10.1093/nar/gkab953, PMID: 34718717 PMC8728281

[63] Piñero J Ramírez-Anguita JM Saüch-Pitarch J Ronzano F Centeno E Sanz F. The DisGeNET knowledge platform for disease genomics: 2019 update. Nucleic Acids Res. (2020) 48:D845–d855. doi: 10.1093/nar/gkz1021, PMID: 31680165 PMC7145631

[64] Safran M Rosen N Twik M BarShir R Stein TI Dahary D. The GeneCards suite In: Abugessaisa I Kasukawa T, editors. Practical Guide to Life Science Databases. Springer Nature Singapore: Singapore (2021). 27–56.

[65] Chen T Zhang H Liu Y Liu YX Huang L. EVenn: easy to create repeatable and editable Venn diagrams and Venn networks online. J Genet Genomics. (2021) 48:863–6. doi: 10.1016/j.jgg.2021.07.007, PMID: 34452851

[66] Otasek D Morris JH Bouças J Pico AR Demchak B. Cytoscape automation: empowering workflow-based network analysis. Genome Biol. (2019) 20:185. doi: 10.1186/s13059-019-1758-4, PMID: 31477170 PMC6717989

[67] Xiong G Wu Z Yi J Fu L Yang Z Hsieh C. ADMETlab 2.0: an integrated online platform for accurate and comprehensive predictions of ADMET properties. Nucleic Acids Res. (2021) 49:W5–w14. doi: 10.1093/nar/gkab255, PMID: 33893803 PMC8262709

[68] Szklarczyk D Gable AL Nastou KC Lyon D Kirsch R Pyysalo S. The STRING database in 2021: customizable protein-protein networks, and functional characterization of user-uploaded gene/measurement sets. Nucleic Acids Res. (2021) 49:D605–d612. doi: 10.1093/nar/gkaa1074, PMID: 33237311 PMC7779004

[69] DennisGJrShermanBTHosackDAYangJGaoWLaneHC. DAVID: database for annotation, visualization, and integrated discovery. Genome Biol. (2003) 4:P3. doi: 10.1186/gb-2003-4-5-p312734009

[70] The bioinformatics online platform (n.d.). Available at: http://www.bioinformatics.com.cn/ (Accessed September 24, 2022).

[71] Burley SK Bhikadiya C Bi C Bittrich S Chen L Crichlow GV. RCSB protein data Bank: celebrating 50 years of the PDB with new tools for understanding and visualizing biological macromolecules in 3D. Protein Sci. (2022) 31:187–208. doi: 10.1002/pro.4213, PMID: 34676613 PMC8740825

[72] Kim S Chen J Cheng T Gindulyte A He J He S. PubChem in 2021: new data content and improved web interfaces. Nucleic Acids Res. (2021) 49:D1388–d1395. doi: 10.1093/nar/gkaa971, PMID: 33151290 PMC7778930

[73] Eberhardt J Santos-Martins D Tillack AF Forli S. AutoDock Vina 1.2.0: new docking methods, expanded force field, and Python bindings. J Chem Inf Model. (2021) 61:3891–8. doi: 10.1021/acs.jcim.1c00203, PMID: 34278794 PMC10683950

[74] Lipinski CA. Lead-and drug-like compounds: the rule-of-five revolution. Drug Discov Today Technol. (2004) 1:337–41. doi: 10.1016/j.ddtec.2004.11.007 24981612

[75] Xu WQ Qin XM Liu YT. Mechanism of Huangqi Jianzhong decoction in treating chronic atrophic gastritis based on network pharmacology. Chin Tradit Herb Drug. (2018) 49:3550–61. doi: 10.7501/j.issn.0253-2670.2018.15.012

[76] Lim EJ Lee JS Lee EJ Jeong SJ Park HY Ahn YC. Nationwide epidemiological characteristics of chronic fatigue syndrome in South Korea. J Transl Med. (2021) 19:502. doi: 10.1186/s12967-021-03170-0, PMID: 34876158 PMC8650266

[77] Hulme K Safari R Thomas S Mercer T White C Van der Linden M. Fatigue interventions in long term, physical health conditions: a scoping review of systematic reviews. PLoS One. (2018) 13:e0203367. doi: 10.1371/journal.pone.0203367, PMID: 30312325 PMC6193578

[78] Zeng M Qi L Guo Y Zhu X Tang X Yong T. Long-term Administration of Triterpenoids from Ganoderma lucidum mitigates age-associated brain physiological decline via regulating sphingolipid metabolism and enhancing autophagy in mice. Front Aging Neurosci. (2021) 13:628860. doi: 10.3389/fnagi.2021.628860, PMID: 34025387 PMC8134542

[79] Wang HY Wang YH Jia YS Du HY. Qualitavive and quantitative Determ Ination of quercetin in Eugenia Caryophllata Thunb. Acta Acad Med Nei Mongol. (2008) 000:534–7. doi: CNKI:SUN:NMYX.0.2008-S1-015

[80] Chen X Liang D Huang Z Jia G Zhao H Liu G. Anti-fatigue effect of quercetin on enhancing muscle function and antioxidant capacity. J Food Biochem. (2021) 45:e13968. doi: 10.1111/jfbc.13968, PMID: 34651301

[81] Liu GP. Effect of food grade quercetin powder on maximal oxygen uptake and endurance of the body. Genom Appl Biol. (2020) 39:6. doi: CNKI:SUN:GXNB.0.2020-01-046

[82] Jan R Khan M Asaf S Lubna Asif S Kim KM. Bioactivity and therapeutic potential of Kaempferol and quercetin: new insights for plant and human health. Plants (Basel). (2022) 11:2623. doi: 10.3390/plants11192623 36235488 PMC9571405

[83] Saw CL Guo Y Yang AY Paredes-Gonzalez X Ramirez C Pung D. The berry constituents quercetin, kaempferol, and pterostilbene synergistically attenuate reactive oxygen species: involvement of the Nrf2-ARE signaling pathway. Food Chem Toxicol. (2014) 72:303–11. doi: 10.1016/j.fct.2014.07.038, PMID: 25111660

[84] Choi W Kim HS Park SH Kim D Hong YD Kim JH. Syringaresinol derived from *Panax ginseng* berry attenuates oxidative stress-induced skin aging via autophagy. J Ginseng Res. (2022) 46:536–42. doi: 10.1016/j.jgr.2021.08.003, PMID: 35818428 PMC9270644

[85] Kim J Lee SH Cho M Lee JY Choi DH Lee HY. Small molecule from natural phytochemical mimics dietary restriction by modulating FoxO3a and metabolic reprogramming. Adv Biosyst. (2020) 4:e1900248. doi: 10.1002/adbi.201900248, PMID: 32558394

[86] Jin SY Li RS Shen BD Bai JX Xu PH Dai L. Lignans-rich extract from Herpetospermum caudigerum alleviate physical fatigue in mice. Chin J Integr Med. (2016) 22:840–5. doi: 10.1007/s11655-016-2254-2, PMID: 27783320

[87] Li E Yan R Yan K Huang R Zhang R Wen Y. Erxian decoction inhibits apoptosis by activating Akt1 and repairs spinal cord injury in rats. Heliyon. (2022) 8:e11279. doi: 10.1016/j.heliyon.2022.e11279, PMID: 36387579 PMC9641199

[88] Wachira J. Morphological alterations of CAD cells overexpressing AKT1. Microsc Microanal. (2020) 26:1354–8. doi: 10.1017/s1431927620017821, PMID: 36237516 PMC9555227

[89] Ha JM Jin SY Lee HS Kum HJ Vafaeinik F Ha HK. Akt1-dependent expression of angiopoietin 1 and 2 in vascular smooth muscle cells leads to vascular stabilization. Exp Mol Med. (2022) 54:1133–45. doi: 10.1038/s12276-022-00819-8, PMID: 35931736 PMC9440121

[90] Shinmura K. Effects of caloric restriction on cardiac oxidative stress and mitochondrial bioenergetics: potential role of cardiac sirtuins. Oxidative Med Cell Longev. (2013) 2013:528935. doi: 10.1155/2013/528935, PMID: 23577224 PMC3614061

[91] Chen X Huang J Hu Z Zhang Q Li X Huang D. Protective effects of dihydroquercetin on an APAP-induced acute liver injury mouse model. Int J Clin Exp Pathol. (2017) 10:10223–32. PMID: 31966356 PMC6965750

[92] Macedo D Jardim C Figueira I Almeida AF McDougall GJ Stewart D. (poly)phenol-digested metabolites modulate alpha-synuclein toxicity by regulating proteostasis. Sci Rep. (2018) 8:6965. doi: 10.1038/s41598-018-25118-z, PMID: 29725038 PMC5934470

[93] Zemanovic S Ivanov MV Ivanova LV Bhatnagar A Michalkiewicz T Teng RJ. Dynamic phosphorylation of the C terminus of Hsp70 regulates the mitochondrial import of SOD2 and redox balance. Cell Rep. (2018) 25:2605–2616.e7. doi: 10.1016/j.celrep.2018.11.015, PMID: 30485823 PMC6377235

[94] Oh HA Kim DE Choi HJ Kim NJ Kim DH. Anti-fatigue effects of 20(S)-Protopanaxadiol and 20(S)-Protopanaxatriol in mice. Biol Pharm Bull. (2015) 38:1415–9. doi: 10.1248/bpb.b15-00230, PMID: 26328499

[95] Ku BM Jung HA Sun JM Ko YH Jeong HS Son YI. High-throughput profiling identifies clinically actionable mutations in salivary duct carcinoma. J Transl Med. (2014) 12:299. doi: 10.1186/s12967-014-0299-6, PMID: 25343854 PMC4216375

[96] Zhou X Xie S Wu S Qi Y Wang Z Zhang H. Golgi phosphoprotein 3 promotes glioma progression via inhibiting Rab5-mediated endocytosis and degradation of epidermal growth factor receptor. Neuro-Oncology. (2017) 19:1628–39. doi: 10.1093/neuonc/nox104, PMID: 28575494 PMC5716177

[97] Kim MJ Choi WG Ahn KJ Chae IG Yu R Back SH. Reduced EGFR level in eIF2α PhosphorylationDeficient hepatocytes is responsible for susceptibility to oxidative stress. Mol Cells. (2020) 43:264–75. doi: 10.14348/molcells.2020.2197, PMID: 32150794 PMC7103887

[98] Chen L Sun L Lang Y Wu J Yao L Ning J. Fast-track surgery improves postoperative clinical recovery and cellular and humoral immunity after esophagectomy for esophageal cancer. BMC Cancer. (2016) 16:449. doi: 10.1186/s12885-016-2506-8, PMID: 27401305 PMC4940721

[99] Pearson NA Packham JC Parsons H Haywood KL. Quality and acceptability of patient-reported outcome measures used to assess fatigue in axial spondyloarthritis (axSpA): a systematic review (protocol). Syst Rev. (2018) 7:116. doi: 10.1186/s13643-018-0777-7, PMID: 30086791 PMC6081943

[100] Vollbracht C Kraft K. Oxidative stress and hyper-inflammation as major drivers of severe COVID-19 and long COVID: implications for the benefit of high-dose intravenous vitamin C. Front Pharmacol. (2022) 13:899198. doi: 10.3389/fphar.2022.899198, PMID: 35571085 PMC9100929

[101] Li Y Li Y Fang Z Huang D Yang Y Zhao D. The effect of Malus doumeri leaf flavonoids on oxidative stress injury induced by hydrogen peroxide (H(2)O(2)) in human embryonic kidney 293 T cells. BMC Complement Med Ther. (2020) 20:276. doi: 10.1186/s12906-020-03072-6, PMID: 32917204 PMC7488428

[102] Vollbracht C Kraft K. Feasibility of vitamin C in the treatment of post viral fatigue with focus on long COVID, based on a systematic review of IV vitamin C on fatigue. Nutrients. (2021) 13:1154. doi: 10.3390/nu13041154 33807280 PMC8066596

[103] Yang Y Liu H Zhao Y Geng C Chao L Hao A. Grim-19 deficiency promotes decidual macrophage autophagy in recurrent spontaneous abortion. Front Endocrinol (Lausanne). (2022) 13:1023194. doi: 10.3389/fendo.2022.1023194, PMID: 36387896 PMC9641028

[104] Lei H Xu J Cheng LJ Guo Q Deng AM Li YS. An increase in the cerebral infarction area during fatigue is mediated by il-6 through an induction of fibrinogen synthesis. Clinics (Sao Paulo). (2014) 69:426–32. doi: 10.6061/clinics/2014(06)10, PMID: 24964308 PMC4050324

[105] Naciute M Mieliauskaite D Rugiene R Nikitenkiene R Jancoriene L Mauricas M. Frequency and significance of parvovirus B19 infection in patients with rheumatoid arthritis. J Gen Virol. (2016) 97:3302–12. doi: 10.1099/jgv.0.000621, PMID: 27902343 PMC5203673

[106] Rich T Zhao F Cruciani RA Cella D Manola J Fisch MJ. Association of fatigue and depression with circulating levels of proinflammatory cytokines and epidermal growth factor receptor ligands: a correlative study of a placebo-controlled fatigue trial. Cancer Manag Res. (2017) 9:1–10. doi: 10.2147/cmar.S115835, PMID: 28203105 PMC5295802

[107] Wang J Ma X Zhang Q Chen Y Wu D Zhao P. The interaction analysis of SNP variants and DNA methylation identifies novel methylated pathogenesis genes in congenital heart diseases. Front Cell Dev Biol. (2021) 9:665514. doi: 10.3389/fcell.2021.665514, PMID: 34041244 PMC8143053

[108] Yin XX Zheng XR Peng W Wu ML Mao XY. Vascular endothelial growth factor (VEGF) as a vital target for brain inflammation during the COVID-19 outbreak. ACS Chem Neurosci. (2020) 11:1704–5. doi: 10.1021/acschemneuro.0c00294, PMID: 32485101

[109] Urbán-Solano A Flores-Gonzalez J Cruz-Lagunas A Pérez-Rubio G Buendia-Roldan I Ramón-Luing LA. High levels of PF4, VEGF-A, and classical monocytes correlate with the platelets count and inflammation during active tuberculosis. Front Immunol. (2022) 13:1016472. doi: 10.3389/fimmu.2022.1016472, PMID: 36325331 PMC9618821

[110] Shang J He Q Chen Y Yu D Sun L Cheng G. miR-15a-5p suppresses inflammation and fibrosis of peritoneal mesothelial cells induced by peritoneal dialysis via targeting VEGFA. J Cell Physiol. (2019) 234:9746–55. doi: 10.1002/jcp.27660, PMID: 30362573

[111] Liu Y Fu H Zuo L. Anti-inflammatory activities of a new VEGF blocker. Conbercept Immunopharmacol Immunotoxicol. (2021) 43:594–8. doi: 10.1080/08923973.2021.1959608, PMID: 34402367

[112] Han R Yu Y Zhao K Wei J Hui Y Gao JM. Lignans from *Eucommia ulmoides* Oliver leaves exhibit neuroprotective effects via activation of the PI3K/Akt/GSK-3β/Nrf2 signaling pathways in H(2)O(2)-treated PC-12 cells. Phytomedicine. (2022) 101:154124. doi: 10.1016/j.phymed.2022.154124, PMID: 35487038

[113] Liu M Zhao G Zhang D An W Lai H Li X. Active fraction of clove induces apoptosis via PI3K/Akt/mTOR-mediated autophagy in human colorectal cancer HCT-116 cells. Int J Oncol. (2018) 53:1363–73. doi: 10.3892/ijo.2018.4465, PMID: 30015913

[114] Deng X Xing X Sun G Xu X Wu H Li G. Guanxin Danshen formulation protects against myocardial ischemia reperfusion injury-induced left ventricular remodeling by upregulating estrogen receptor beta. Front Pharmacol. (2017) 8:777. doi: 10.3389/fphar.2017.00777, PMID: 29163163 PMC5671976

[115] Sun L Xu G Dong Y Li M Yang L Lu W. Quercetin protects against lipopolysaccharide-induced intestinal oxidative stress in broiler chickens through activation of Nrf2 pathway. Molecules. (2020) 25:1053. doi: 10.3390/molecules25051053, PMID: 32110995 PMC7179181

[116] Park SE Sapkota K Kim S Kim H Kim SJ. Kaempferol acts through mitogen-activated protein kinases and protein kinase B/AKT to elicit protection in a model of neuroinflammation in BV2 microglial cells. Br J Pharmacol. (2011) 164:1008–25. doi: 10.1111/j.1476-5381.2011.01389.x, PMID: 21449918 PMC3195922

[117] Oh JH Joo YH Karadeniz F Ko J Kong CS. Syringaresinol inhibits UVA-induced MMP-1 expression by suppression of MAPK/AP-1 signaling in HaCaT keratinocytes and human dermal fibroblasts. Int J Mol Sci. (2020) 21:3981. doi: 10.3390/ijms21113981 32492931 PMC7312901

